# The social construction of genomics and genetic analysis in ocular diseases in Ibadan, South-western Nigeria

**DOI:** 10.1371/journal.pone.0278286

**Published:** 2022-12-01

**Authors:** Olusola Olawoye, Kabiru K. Salami, Abolaji Azeez, Precious Adebola, Tarela Sarimiye, John Imaledo, Tony Realini, Michael A. Hauser, Adeyinka Ashaye

**Affiliations:** 1 Department of Ophthalmology, Faculty of Clinical Sciences, College of Medicine, University of Ibadan, Ibadan, Nigeria; 2 Department of Ophthalmology, University College Hospital Ibadan, Ibadan, Nigeria; 3 Department of Sociology, Faculty of the Social Sciences, University of Ibadan, Ibadan, Nigeria; 4 Department of Health Promotion and Education, Faculty of Public Health, College of Medicine, University of Ibadan, Ibadan, Nigeria; 5 Department of Ophthalmology and Visual Sciences, School of Medicine, West Virginia University, Morgantown, West Virginia, United States of America; 6 Department of Medicine, Duke University School of Medicine, Duke University, Durham, North Carolina, United States of America; 7 Department of Ophthalmology, Duke University School of Medicine, Duke University, Durham, North Carolina, United States of America; La Trobe University - Melbourne Campus: La Trobe University, AUSTRALIA

## Abstract

Genomics, an emerging field to improve public health practice, has potential benefits to understanding ocular diseases. This study explored the social construction of genomics in ocular diseases in the blind community in Ibadan, Nigeria, through two focus group discussions and twelve in-depth interview sessions conducted among people living with ocular disorders. The data were thematic and content-analysed. Although the participants had limited knowledge about ocular diseases, genomics, and their nexus, they maintained a positive attitude toward its potential benefits. This informed their willingness to participate in genomics testing for ocular diseases. The participants preferred saliva-based sample collection over blood-based, and expressed concern for the procedure and accrued benefits of genomics studies. Thus, public sensitisation about ocular diseases and client-centred genomics testing procedures should be engendered.

## Introduction

The emerging field of genomics offers the potential for greater understanding of the genetic basis for many disorders, including those commonly associated with vision loss and blindness [1]. The technical and technological aspects of genomic research are unfamiliar to many Africans, as are the nature and impact of common ocular disorders on visual function [2, 3]. The current knowledge of genetics and genomics is still very low in Africa despite its potential to reduce the burden of many genetic diseases. Such unfamiliarity poses a significant barrier to the participation of many Africans in genomic research [4]. Genomic research is important in understanding the genetic basis of disease, illness and treatment response [5]. Genomic research can facilitate individualized patient care to improve early diagnosis of diseases which may lead to improved management and follow up care [6]. With respect to ocular disease, genomic investigations into the underlying causes of age-related macular degeneration (AMD) uncovered the role of vascular endothelial growth factor (VEGF), which in turn led to the highly effective use of anti-VEGF antibody injections to treat AMD [7]. Replicating this success in the treatment of glaucoma requires detailed studies of the genomic basis of the disease, especially in the understudied and underserved African populations.

Several factors have been reported to be responsible for the high prevalence and incidence of preventable blindness in Sub-Saharan Africa. Some of these include cost, lack of knowledge, as well as cultural and social barriers [8]. Ofosu et al. [3] also reported in their study in Ghana that lack of awareness affects health seeking behaviour and eye care service delivery. They reported that although participants in their survey knew that cataract could lead to poor vision, less than a fifth (18.6%) of them could relate cataract with blindness. In another study carried out in Osun State of Nigeria, Isawunmi et al. [9] reported that only 15.8% of the respondents in an adult rural population had ever heard of glaucoma. In Bangladesh, it was reported in a study that over half of the participants in their study did not know that their vision loss could be treated [2]; this was more pronounced amongst people with low socioeconomic status and older people. In India, Senthilkumar et al. [10] reported that poor knowledge of diseases and their symptoms among parents of children was responsible for their poor health seeking behaviour.

Awareness and genomic literacy are important aspects of managing genetic diseases. Available knowledge in the field of genomics has been mostly from studies conducted among populations of European descent [11]. About a decade ago however, the National Institutes of Health (NIH) and the Wellcome trust launched the H3Africa (Human Heredity and Health in Africa) initiative to facilitate genomic research with the aim of improving the health of African populations. The goal of H3Africa was to promote a contemporary research approach to the study of genomics and environmental determinants of common diseases in African populations [11]. The genomic research under this initiative had challenges related to unawareness and lack of understanding of genomics research which affected patient participation especially. One major technique and solution to address this knowledge gap is community engagement to identify and address community members’ misconceptions of genomics research. Improved awareness and knowledge of the relevance and potential impact of genomic research to human disease in the community may lead to improvement in recruitment and participation in genomic studies.

Genomics in public health represents a multidisciplinary approach to the effective and responsible use of genome-based knowledge and technologies to improve population health [12]. Globally, the current challenge facing genetic science is to fully decipher its contribution to human disease and health conditions [13]. The positive contributions of genomics have been explored in various fields. For example, Duquette [14] observed that genomics would be relevant to public health dentistry. Also, in cancer research, genomics remains the major tool for understanding mutations that predispose individuals to the disease.

The global public health community has not fully adopted actionable genomics possibly due to the newness or complexities of the field [15–18]. Boersma and Gremmen [19] observed that unfavourable attitudes toward human genomics emanated from labelling genomics as genetic manipulation. For instance, in a review of knowledge and attitudes towards human genomics and testing, Odukoya et al. [20] reported that knowledge about genomics in Nigeria consistently emanated from online platforms and more often focused on sickle cell disease. Cultural beliefs in different countries influence the acceptance of genomics testing. In developed settings, genomics research is growing and appreciable efforts are underway even in ocular diseases; however, insufficient attention has been given to genomics and ocular disease in Africa as a whole and Nigeria in particular.

The Eyes of Africa: The Genetics of Blindness is a collaborative research program sponsored by the National Institutes of Health to identify new susceptibility genes for primary open angle glaucoma (POAG) and to develop a strategy for community engagement in Sub Saharan Africa (SSA). POAG is a complex multifactorial disease with a largely unknown etiology, although both genetic and environmental factors have been established. While significant progress has been made in identifying risk factors for glaucoma in European ancestry individuals [21], the genetic risk factors for individuals of African ancestry remain largely unknown [22]. The identification of novel genetic risk factors for glaucoma is one of the primary goals of the Eyes of Africa project. No genetic test currently exists that can accurately measure an individual’s risk of glaucoma. It is our hope that knowledge of genetic risk factors in African ancestry individuals will enable the development of such tests in the future. Although 90% of patients with visual impairment live in Africa and other low and middle income countries [23, 24], African populations still have the least access to eye care [25–27] and also have poor knowledge of eye care [28].

The community engagement component of the Eyes of Africa project is intended to improve the understanding of genetics and genomics in the population, and this paper describes the state of knowledge among the population before interventions. In this paper, we explore the knowledge, attitude and perception of genomic testing among persons living with ocular diseases in Nigeria.

## Materials and methods

### Study design

This qualitative study utilised in-depth interviews (IDIs) and focus group discussions (FGDs) to generate data from members of the Nigeria Association of the Blind (NAB), Oyo State branch, and the blind residents of Orita-Aperin community, respectively. The study protocol was reviewed and approved by the Ethics Committee of the University of Ibadan/ University College Hospital Research Ethics Committee with study protocol number UI/EC/17/0394. Before the onset of the study, two advocacy visits were made to Orita-Aperin community and the members of the blind association during their monthly meeting. The first visit was made to the blind association, the purpose of which was to inform members of the study, to explain its purpose, the duration of the interview, and the potential risks and benefits of participating in the study. Written consent was obtained from the association leadership to conduct this study following a future monthly meeting. The second visit was made to the community’s traditional leadership where the community head was also duly informed about the study. Consent was also obtained from the head to conduct the study in the community among the blind. All participants gave verbal consent before being included in the study. The principles governing human research were adhered to by the researchers. Participants were fully informed about the study, and they were assured of the confidentiality of their responses, hence the rights and integrity of the participants were ensured and protected. The participants were not forced or coerced to participate in this study. Finally, the participants were informed that they were free to withdraw from the study without any consequence. Permission to audio-record the discussions and responses was obtained verbally from the participants.

### Study setting

Orita-Aperin is a densely populated urban low-resource community located in the Ibadan South East Local Government Area of Oyo State. The majority of its residents are traders, artisans and retired civil servants with a long history of adhering to traditional customs. The members of the Oyo State branch of NAB hold a monthly meeting at Orita-Aperin Community, a location selected for its proximity to where many of their members teach at the Omoyeni Special Basic School for the Blind. Membership includes male and female adults aged 18 years and older. A review of the association’s register prior to the study showed a population of about 100 registered blind people. The association, which receives members from all parts of the city, has a clearly stated vision and mission, boldly printed in most of their documents, to promote the rights of the blind and partially sighted persons in Nigeria through advocacy, capacity building and partnership, while their vision statement reads ‘A world of total inclusion and equal opportunities for all’.

### Participants

For the IDIs, all members of NAB present at the meeting on the study date were informed again about what the study entailed, including the purpose of the study, the study process, the duration of the interview, and the need to audio-record the interviews. The IDI participants were interviewed from among those who indicated interest to participate, until a representative sample was obtained. For the FGDs, consented NAB members directed researchers to other potential participants whom the researchers then contacted through personal visits and sought their consent to participate. All participants who were selected to participate in the FGDs were informed and invited to a location within the community. The FGD participants were provided with transport fees to the venue of the discussion and refreshments were provided upon arrival.

### Techniques of data collection

The interviews and discussions lasted approximately forty-five minutes each. Interviews and discussions were conducted in both English and Yoruba, the local dialect, by trained interviewers using standardised/pretested interview and FGD guides. These instruments were designed in the English language but translated to Yoruba, the local language. Both IDI and FGD guides were subjected to validity and reliability checks for appropriateness through review by experts in the field of ophthalmology, specialists in ocular disorders, and an expert in the behavioural sciences field. Field staff with experience in social science research were trained on the use of the interview and discussion guides for one day. On second day, field staff role played the roles of interviewers, moderators, and note takers, as well as participants. Thereafter, field staff pretested the instruments and field procedures to persons living with ocular disorders in another community. At the end, lessons learnt were incorporated into the final techniques of data collection.

### Data analysis

Audio recordings of the IDI and FGD sessions were transcribed and labelled appropriately devoid of participants’ identifiers, while field notes were duly subjected to further validation to ensure that content and meanings were preserved. After transcription, data were reviewed and edited to ensure proper interpretation and construction of accurate meaning. The transcribed notes were processed using NVIVO version 12 Pro (www.qsrinternational.com/nvivo-qualitative-data-analysis-software/home) software designed for qualitative data analysis. Both deductive and inductive approaches were employed in coding of the data. The deductive coding process involves the identification of themes with relevance specific to the research focus, research questions, the research context, and the framework analysis [29–31]. This approach allows data to be both described and interpreted for meaning [32–35]. Narratives from interviews were transcribed verbatim and a thematic analysis was undertaken [36–38] through the framework analysis approach [29, 30, 39]. The framework enabled both deductive and inductive theme identification using an exploratory “grounded” approach [40, 41] through two phases comprising five main steps. The first phase consisted of managing the data cropped from all the transcripts, in three steps: familiarization with the data, development of a thematic/coding framework, and indexing. The second phase consisted of data explanation including data charting and data mapping. Themes emerging based on patterns of results as reflected in similar codes and quotations were further explored by the research team, based on major points of agreements, contrasting views, and striking and salient points that were made by the participants. The participants’ languages, ideas and opinions were carefully examined, systematically categorized, and recurrent themes noted. Coding was conducted by one of the authors AA, an experienced analyst and sociologist, trained in data analytics, who has participated in many courses on qualitative analysis including one on data displacement: Qualitative data Analysis using NVivo 12 Pro.

Comparisons and trends were then identified using data matrices as suggested by Maxwell [42]. Both phases enhanced theory-driven and data-driven analysis [38, 43]. Thematic analysis is appropriate from the perspective of Braun and Clarke [38] ‘when investigating an under-researched area or while working with participants whose views on the topic are not known’. Analysis of genomics in ocular disorders is one core ethical area that is not fully engaged in literature. This analysis, as well as coding, also benefitted from analytical rigour and guides derived from other experts in behavioural and ophthalmological studies [44]; hence queries were raised and cleared with insights into statements and data with the hope of identifying the patterns within the whole data set. The quotes that were used to substantiate the narratives emerged from the data and were referenced and/or attributed to identifiers by category of data source (IDI, FGD), gender (Male, Female) and individual discussant number in the group discussion (D1–D6). The overall analysis is presented in the results and discussion section.

## Results and discussion

### Participants demographic data

Twelve IDI participants comprising a representative sample were selected and interviewed from among those who indicated interest to participate. Participants were males and females from 29 and 52 years of age. Seven were 40–50 years of age, four were 29–39 years of age, while one was fifty-two years of age. Nine participants were civil servants (eight were school teachers and one worked with the State secretariat). One each was engaged in hand craft, a student and unemployed as shown in Table 1. Two FGDs were conducted among male and female groups separately. Membership size of each group was six. Discussants were residents in Orita-Aperin community, and all were blind, with their age ranging between 19 and 63 years. All the FGDs discussants were engaged in economic activities through which they earned their income (as shown in Table 2).

**Table 1:** Metadata of in-depth interview participants.

S/N	Pseudo Name of Participants	Gender	Age (Years)	Occupation
1	IDI_1	Female	38	Teaching
2	IDI_2	Female	50	Teaching
3	IDI_3	Female	40	Teaching
4	IDI_4	Female	46	Teaching
5	IDI_5	Female	48	Teaching
6	IDI_6	Female	44	Teaching
7	IDI_7	Male	45	Civil Service
8	IDI_8	Male	29	Student
9	IDI_9	Male	36	Teaching
10	IDI_10	Male	43	Hand craft
11	IDI_11	Male	30	Unemployed
12	IDI_12	Male	52	Teaching

**Table 2:** Metadata of members of focus group discussion.

S/N	Pseudo Name of Discussants	Groups	Sex	Age (years)	Occupation
1	Discussant 1	Group 1	Male	28	Radio Host
2	Discussant 2	Group 1	Male	39	Teaching
3	Discussant 3	Group 1	Male	41	Teaching
4	Discussant 4	Group 1	Male	41	Teaching
5	Discussant 5	Group 1	Male	50	Teaching
6	Discussant 6	Group 1	Male	63	Retired Teacher
7	Discussant 1	Group 2	Female	19	Student
8	Discussant 2	Group 2	Female	33	Petty Trading
9	Discussant 3	Group 2	Female	39	Hand Craft
10	Discussant 4	Group 2	Female	45	Hand Crafts
11	Discussant 5	Group 2	Female	46	Teaching
12	Discussant 6	Group 2	Female	48	Teaching

### Knowledge of genetics and ocular disorders

The analysis of participants’ narratives showed various levels of knowledge about the genetic basis of disease etiology. The participants differentiated sexually transmitted diseases from gene-driven diseases. The participants asserted belief in the tendency for individuals to inherit diseases from parents, of which ocular disorders were included. Other conditions identified as inheritable from parents included substance addiction, high blood pressure, speech impairment, deafness, epilepsy, oncological and cognitive/developmental disorders. Also, participants attributed some behavioural traits to gene-inheritance from parents, while some participants attributed their ocular disorders to their family background, even though none of their biological parents had similar disorders. An emerging expression from the participants’ narratives related to the possibility of both intra- and inter-generational dimensions of disease inheritance, along maternal or paternal paths.

Analysis of narratives in this study also showed the belief that most inherited diseases are passed mother-to-child. The rationale for this conclusion is embedded in the relationship that mothers share with their child during conception through birth and upbringing. However, some participants expressed the need to understand why their immediate parents did not have ocular conditions, since inherited diseases were perceived to be transmitted through breastfeeding and blood contact. The potential sources of information about ocular diseases included radio programs, television programs, hospitals, household members and through personal experiences. Most participants reportedly knew at least one person with an ocular disorder, while glaucoma was considered to be a common eye disorder among people with eye problems. This may be related to heredity as indicated in this study, and corroborates with other studies [1].

In one narrative, a female participant stated that ‘……*it happens like that*, *it is true*, *that the father or mother could have it*, *and it could spread to the child too; so it has been in existence for a long time since the days of our forefathers’*, while another participant attributed his own perception to religious perspective as shown in an excerpt of his narrative:

Bible didn’t tell us we can inherit any disease. The disease that one can inherit include this my condition [glaucoma]; I am someone that can’t see very well as I am. So if a father or mother can’t see very well, to the extent that the disease resides in the blood of the parents, then the child can inherit it from them (/IDI/Male).

### Experiential causes of ocular diseases

As most participants had acquired rather than congenital ocular disorders, the circumstances that preceded the loss of sight and the dynamics of eventual visual impairment of the participants were analysed. Participants described five typologies of causes: natural, supernatural, preternatural, accidental and self-inflicted ocular diseases. Participants clearly emphasised that the circumstances that brought about the ocular disorder and their nature influenced the health-seeking behaviour and the pathways of persons living with ocular disorders. All participants purportedly sought ophthalmic care as a last resort. Also, the loss of vision was reportedly not limited to a particular age.

However, a series of explanations of the causation typologies emerged from the narratives of the participants. For instance, some participants alluded to the potency of metaphysical power as being responsible for blindness. In the male group, a discussant expressed that “*visual impairment is a metaphysical tool to preclude glorious individuals from achieving their predestined greatness*.” Another discussant exemplified the same position that “*for example*, *someone can be cursed and it would result into blindness*. *Such metaphysical tool was not limited to blindness alone*, *but also includes disease like deafness*.” The supernatural explanation of blindness was attributed to ‘*god’s anger’* against the individual, according to a female discussant. Similarly, accidental causation was attributed to physical injury to the eyes which results in blindness, while self-inflicted blindness occurs when an affected individual delays treating the ocular disease at the inception, or due to drug abuse by pregnant women, or battering of pregnant women. In a narrative, heredity as a cause was exemplified by a female discussant:

What I know about inherited blindness? For a family that I know, blindness is in one of the parents. They also inherited it from their parents such that out of five siblings, only one was not blind, others were blind. You know something like that is hereditary. There is a child in this school, they said that’s how he was born; that he was born blind. It occurred in the bible too (FGD/Female/D5).

### Acceptance of genomics testing

The analysis of genomics testing and ocular disorders shows that participants possessed very limited prior knowledge about genomics and its potential benefits in ocular sciences. Although more recent studies have expanded the discussions on the relevance of genomics science [14, 16, 18], little attention has been paid to how genomics could be of help in the prevention or treatment of ocular diseases. Participants in this study, having been told of the potential nexus between genomics and ocular disease, subsequently showed a keen interest in genomics and expressed their willingness to participate in genomics research. Most participants asserted the opinion that genomics, as current scientific developments, would reduce the incidence of ocular diseases. This is similar to a recent observation that genomics would improve public health [12]. A male participant felt that the ‘*scientific move*’ should be labelled as “*the sustainability of human development*.” However, participants also thought that those who are not well informed might not participate in genomics research. Consequently, the little or no information available to the people about the genomics testing in Nigeria would inform their interpretation of process. This was observed by a participant, *“…*. *for example*, *some people might suspect or accuse the researchers of being fetish*, *which would disrupt the continuity of the genomics research*.” The participants assured that once people are properly informed about genomics, they would be willing to participate. From a narrative, a participant expressed his support for genomics research as follows:

I will definitely support it because I have series of plans concerning issues like this; to help do something to prevent blindness or treat the disease. And I won’t just donate my blood, if I am financially buoyant, I will also give money to support the research that is being carried out. I am saying I will donate my blood and other things that are required to conduct the research (IDI/MALE).

Concerning the possibility of treatment for inherited diseases, participants also expressed the belief that disorders of heredity, such as ocular diseases, could be treated, and cured, hence reduce their prevalence and incidence in the community. The content analysis of the participants’ narratives about trust of genomics testing indicates that genomics testing in Nigeria would engender positive changes in ocular diseases, while participants expressed confidence that with the present level of advancement in science, ocular disease could be prevented.

### Collection of samples for testing genomics in blindness: Knowledge, belief and preference

Despite declaration of positive support for genomics research by participants in this study, there are indications that people living with visual impairment still have limited knowledge about blood sample donation for genomics testing. Clearly, genomics in ocular disorders was a relatively new concept to the participants. For instance, a female participant perceived the small quantity of blood to be collected as “*sufficient enough to damage health or affect people’s wellbeing negatively*.” Participants were also concerned about access issues including availability of adequate services, competent specialists to collect the samples, who bears the cost of recuperating if donation of samples cause problem, and the relevance of blood samples if the blood testing indicates the presence of ocular disease. Despite the access issues, most participants applauded the collection of blood samples to test for genetic ocular disorders that could result in blindness. The acceptance of genomics in this study hinged on the possible overall benefits the genomics research might bring to ocular diseases. A participant was concerned about how the lack of blood samples would preclude accomplishment of genomics research. Other participants maintained that individuals should be allowed to decide when it comes to blood sample donation. Consequently, donor-centeredness will be important in future genomics research [15–17]. A participant who was favourable to testing expressed his view in the following excerpt:

It is possible for one to inherit a disorder from parents yet one may not know. However, if such person associates themselves with those who are well experienced, they would be advised to go for test, and when he gets to the hospital, they can request for any sample for testing. (IDI/MALE).

An elderly male participant expressed concern about blood sample collection for genomics research, saying that in metaphysical theology, witches and wizards would suck blood; however, narratives from female discussants revealed that such beliefs are fading.

Participants expressed preferences for types of samples to be collected for genomics testing for ocular disorders. Individual preference was guided by any, or all of six factors: the individual’s value attached to a sample, the potential for pain in donating the sample, the volume of the sample, cost of replacing/recuperating from possible side-effects, social construction of the sample, and the nature of the sample. Most participants preferred to donate saliva, followed by the blood donation, while only few would donate faeces. In spite of the conditions that guided the preferences, participants considered ‘convenience’ as the utmost important variable determining choice of sample among their preferences. Donation of saliva was based on “*simple to donate with ease*, *without losing anything*,” while participants that preferred blood sample maintained that “*blood would provide more information about a person than saliva*.” The choice of blood could emanate from the blood samples often collected in hospitals or medical laboratories to carry out tests. Overall, the perceived wellbeing and potential benefit from the genomics research made the participants in this study reveal their preferences for genomics testing. This is contrary to findings by Boersma and Gremmen [19] where negative attitude toward genomics testing was reported in their study. Clearly, proper education about the benefit of genomics remains germane in that context.

### Propensity to donate sample for genomics research: Religion and literacy factors

Religious beliefs may inhibit sample donation. One participant exemplified a religious sect that would not participate in such research, since the doctrine of their sect negates blood transfusion and has reservations about medical activities that require blood. People living with ocular disorders also expressed a level of fear about any procedure that requires donation of bodily fluid especially when there was no forerunner in the genomics research. Although most participants opined that the genomics research initiatives remained plausible, they displayed skepticism in their readiness to participate. From the narrative of a male discussant group, to motivate people to participate “*medical practitioners should conduct basic medical test for them before their eventual participation in the study*. *Such a test would convince them first that this is a serious study; and it would motivate them to participate*.” A discussant in a female FGD session expressed that further benefits accrue to participants of genomics research in the following excerpt:

Giving blood sample for a research is something that is important and very good. It will help someone know the path they would follow if the disease is in their body, so they would monitor it and it won’t propagate in the body. So, they would finish it from the onset. And they would be able to tell their children on the appropriate actions to take at certain age in order to monitor themselves and care for a certain disease that runs in their family blood. So it’s very good (FGD/Female/D2).

However, a male participant pointed out illiteracy as a factor that may likely challenge genomic science in ocular diseases.

### Presentation of genomics research results and feedback communication to the participants

Access to the results of sample testing was also a concern raised by the participants. Every individual who participated in sample screening desired to receive the results, in order to know their health status, and desired to have access to the outcome of the study, since their body fluid was collected for the study. All participants affirmed that their participation in such studies hinged on the belief that each participant would have access to their personal medical information, a perception that was in consonance with a previous study [20] where sharing results was considered important for participation. The narrative from the female discussion group clearly asserted that sharing sample screening results with the participant remains a mutual responsibility of the researcher and the researched:

Ah, it should be collected. If one would not collect the result, then there is no need to donate a blood sample. Anyone that leaves a blood sample is mandated to collect the result of the test so that one would know the solution to the problem. And if it is good, there is no problem (FGD/Female/D6).

There was a clear indication that the participants were aware that the result of the study would benefit other people who have the same health condition, and even the public at large; however, sample donors were expected to benefit before other persons. Monetary reward, as incentive, was asserted by a participant as the first direct benefit to donors for participation in the study. Another discussant also argued assertively that there is no reason to participate in a research study that would not benefit one directly. One of the discussants explained how the immediate yet direct monetary incentive is germane, even when the genomics outcome is not favourable to the donor, saying “…the participant would not lose at both ends”. The discussant continued her explanation in the following excerpt:

… . the thing is that I would be willing to donate my blood sample, but you and I would have an agreement. That’s what it is. I can leave it behind but there should be an agreement on monetary incentive. That if the results come through and does not favour me, I’ll still have something to benefit. You cannot take my blood sample just like that. So, that’s the agreement we would have (FGD/Female/D4).

A previous study showed that participants in genomics studies would not permit, and would not be pleased, that the research team share their information with other persons and professionals [20]. Although the previous study did not disclose the level of anonymity which the researcher promised the study participants in sharing the genomics results, most participants in the current study were comfortable with the sharing of their valid results with other professionals. Generally, the impression is that genomics research outcomes would aid prevention and better management of ocular diseases. In the same vein, a male participant maintained that a research team is allowed to post results online especially when the study participants were duly informed about the information-sharing rights of that research. On the other hand, a female participant cautiously argued that individuals with stigmatized diseases would not want such results to be shared with other persons or professionals. Another participant shared her feelings about sharing of results with other professionals, in the following extract:

I would agree to testing, but I would be happy if they tell me what they found in the test before sharing with other professionals. At least if they cannot take care of me then let me be aware of the steps I’d take on time in order not to ruin my future and that of my children (IDI/Female).

Clearly, the information needs of the study participants should be met. This was evident in one of the IDI sessions. One participant requested to know the personal consequence of his participation in the study. His emphasis was “to know more about ‘after-study-episode’ and tendency to get further treatment if there would be need for the treatment”. The following narratives from a male discussant group also highlight some concerns of a potential participant in genomics research:

Like for me oh, what I would first ask is, if I donate blood sample and the study test shows there’s a disease in my blood, is there any assistance to treat me for the disease that was found? Are you clear with my concern? So, if there would be a treatment then I would agree to participate on the research. However, if there would not be treatment, there’s nothing I can do with the study (FGD/Male/D6).

As important as information needs concerns of the participants, only little evidence [45] indicated successful application of existing eye health information to support interventions that concern community participation to improve quality of care and access to comprehensive eye health services in eye health programs.

### Relevance of findings to future genomics research in Africa

The focus of the community engagement component of the Eyes of Africa project was to improve the understanding of genetics and genomics in the population and to generate adequate community participation. Our results suggest ways to better design genomics studies, not only in the Eyes of Africa project but in other projects and programmes on eye health, ocular diseases, and genomics. Despite a more holistic and integrated commitments at global, regional, and national levels, most of the eye health programs still face unmet needs [28]. The lack of strong community engagement strategies educating the populace on eye diseases is partly due to inadequate numbers of personnel with appropriate competences, leading to poor outreach and consequently poor knowledge of the populace on eye care. These represent serious issues for consideration in future program planning and implementation on eye care.

Future research and programs should provide further clarity on two issues while recruiting study participants. First, that participants insisted that the results of genetic testing were required to know whether the individuals need treatment. This is clearly untrue, as many diagnoses can be made on the basis of clinical evaluations alone and at present, genetic testing does not improve diagnostic accuracy. Hence, study participation and treatment are entirely separate. This should be made clear in the community engagement activities. Second, participants asserted that results should be shared with individuals in an identifiable way. This is an entirely reasonable expectation for an informative and clinically actionable genetic test; however, no such test currently exists for primary open angle glaucoma, which is a complex, multifactorial disease that does not follow a Mendelian inheritance pattern. There is currently no context in which study findings can be interpreted with regard to risk of developing glaucoma. In fact, one of the ultimate goals of this genetic study is the creation of such an informative genetic test. Participants should be informed that the clinical significance of genetic findings is often unknown and unknowable and only aggregate results will be published. This issue is addressed specifically in the Eyes of Africa informed consent document, which includes this statement: “The experimental procedures have been explained and no guarantee has been given about the possible results.” Future community engagement activities can include more detailed explanations of the current lack of informative genetic tests, and our desire to develop such tests through genetic studies such as ours.

The need for these clarifications while recruiting future study participants arises because of the low level of genomics knowledge in this study population. This lack of understanding is neither new nor limited to this study population. Senthilkumar *et al*., [10] reported that their study has also shown that many patients had limited knowledge of ocular diseases. Through a review of published literature, WHO policy documents, and examples of eye and health care interventions in sub-Saharan Africa, du Toit *et al*., [45] found that the impact of unmet eye care needs in sub-Saharan Africa is compounded by barriers to accessing eye care, limited engagement with communities, a shortage of appropriately skilled health personnel, and inadequate support from health systems.

Going forward, it is clearly important for future studies to identify what education should be contemplated to improve understanding and participation in genomics studies. Community consultation is important. Kane, Gerretsen, Scherpbier, Dal Poz, and Dieleman [46] noted that if an intervention did not address an unmet need of the community, it was less likely to be successful. The unmet needs of participants in this study are genuinely low knowledge of genomics and participation in genomics research. Adequate attention should be placed on these unmet needs to institute solutions in order to avoid program failure in community engagement activities. For instance, du Toit, *et al*. [45] noted that failure of many programs has damaged the credibility of community health activities. Factors contributing to failure include a lack of understanding of community work, unrealistic expectations, poor planning and underestimation of the finances, resources, and support required for a successful program [47–50]. These factors are similar to some issues raised in the narratives of this study.

Our study revealed a surprising level of knowledge regarding the genetic basis of ocular disorders, but tempered with many fallacious beliefs such as maternal versus paternal contributions to inheritance, and supernatural/religious underpinnings, among others. Education should recognise the willingness of many to participate in genomics research and address preferences regarding the nature of samples collected, the importance of considering religious beliefs regarding sample types/blood donation, and the belief that one should be tested for something before participation to ensure that the sample is of value to the project. Finally, the education should address the expectation that participants benefit in some tangible way from their participation (monetarily for the sample, with feedback on findings of their personal sample as well as the overall study findings). Clearly, while all these may not apply in all research settings, application of most of these will help in the dynamic [51] design and implementation of future genomics research and programming in Nigeria and SSA. Also, as this study is an in-depth analysis of a specific geographic group within Nigeria, a limitation would be that these findings may or may not extrapolate to other regions/countries with different cultural, political, and economic factors.

## Conclusion

Genomics research is still novel especially in ocular diseases, with promising potentials. Participants appreciated the importance of genomics research in relieving ocular diseases. Knowledge limitations influenced the attitude of the participants towards genomics in ocular sciences. This poses the need to educate people about current developments in the field of genomics and ocular diseases. These education needs should be met through adequate mass and social publicity. Also, the informed consent process must be thorough, adequate and applied diligently by the researchers in the field. The participant’s opinions about anonymity should be sought, understood and respected to engender greater support and participation in genomics research. Similarly, blood sample donors should be assured that blood collection will be handled by a specialist. Unwillingness to participate necessitates the need to further inform the participants in order to build and gain their confidence for genomics research in ocular diseases. The development of actionable genomics procedures in ocular diseases should be sensitive to the needs of the study participants.

## Supporting information

S1 Appendix Metadata of IDI and FGD participants. (ZIP)
