# Supplementary material for: The social construction of genomics and genetic analysis in ocular diseases in Ibadan, South-western Nigeria
Source: PLoS One. 2022 Dec 1;17(12):e0278286. doi: 10.1371/journal.pone.0278286 (PMC9714877; doi:10.1371/journal.pone.0278286)
Supplement: S1 Appendix — (ZIP) [file pone.0278286.s001.zip › Female FGD Transcript.docx]

**Women FGD**

(*I: Interviewer, W: Woman 1-6*)

**Women in background**: Good morning.

**I**: Good morning, hope you are doing fine. The question I want to ask you ma is that, from Woman 1. What do you know about diseases that are inherited?

**W1**: That is found in the blood?

**I**: Yes, and is inherited.

**W1**: Diseases in the blood that are inherited are quite many that are heritable. Like for instance, many of us who are blind may have inherited it. It could be that the lineage had it and so it was inherited when he/she was born. Others also say that hypertension is inherited that some people have it in their lineage so when they have children those children inherit it when they grow up. Or some strange behaviours exhibited and people would say “He inherited it, he got it from home” that’s all I can say about it.

**I**: Thank you Woman 1. Woman 2, what do you know about inherited diseases asides from what Woman 1 has said?

**W2**: There are some diseases that are different from what Woman 1 has said and are also inherited. In some families, mental illnesses could be disturbing them. When they have children those children also suffer from such and people would say “This is person has also started manifesting madness” and others will say “He inherited it from his forefather, they have also exhibited such in the past”. So that’s also a heritable disease that people inherit.

**I**: Thank you Woman 2. Woman 3, do you have an idea of other diseases that are heritable asides from what Woman 1 and 2 have said?

**W3**: The thing is, like hypertension now is heritable. I saw someone who when he was talking – it wasn’t like – er… one of them kind of has a mental illness but his speech isn’t very audible but the rest of them don’t have the illness but their speech isn’t good either. You know, when someone is – the person is not a “stammerer” but their speech is not fluent and that’s how they all speak. It seems it is hereditary. So that’s how I observe it, that’s what I can say about that.

**I**: Thank you Woman 3. Woman 4, do you have any other explanations asides from what Woman 3 has said? Or is there some ailment you want to mention that hasn’t been mentioned?

**W4**: Ehh… like cancer, people call it a hereditary disease. Because some people perhaps someone has had the disease before in their lineage so it could be passed down to another generation and manifest. Or deafness, it could be that in their family someone had been deaf a long time ago, it could happen to some of the children. So that too could be called a heritable disease.

**I**: Thank you Woman 4. Woman 5, do you have any other disease you could mention that you think could be inherited?

**W5**: Mm, all what they have mentioned is all part of it. So I don’t think there are others I know.

**I**: Thank you Woman 5. I want to ask you all if there are other names in your society in which people use to refer to heritable diseases. Maybe there are other names for these words. Like maybe they call it hereditary disease or generative diseases, but maybe there are other names it is called in your society.

**Chorus response:** It is hereditary disease, it’s hereditary disease.

**I**: Okay, thank you. I want to ask Woman 1, do you know anyone who has lost their sight?

**W1**: You mean who doesn’t see anymore?

**I**: Yes ma.

**W1**: I myself, I know, I know a lot of such people.

**I**: Okay, what do you think could have caused it?

**W1**: Ehn, what could have caused it is that for some people it’s a sickness and for some it was inherited. So... and some it could be that it was the work of supernatural forces.

**I**: Okay Woman 1, you said sickness could cause it, what type of sickness do you think could cause it?

**W1**: There is one sickness called measles, and it usually causes it. If it affects a child, it could affect the eyes or the ears.

**I**: Okay thank you ma. Woman 2, I want to ask you, do you know anyone who is a teenager who has lost their sight?

**W2**: Several, there are many that I know well who live in my neighbourhood.

**I**: Do you think that something else could have caused it asides from what Woman 1 has mentioned?

**W2**: Yes if Glaucoma runs in the blood of their family. So it could cause it, they would say its glaucoma that is affecting them.

**I**: Thank you Woman 2. Woman 3, I want to ask you, do you have the belief that it could be caused by supernatural forces? Maybe blindness could be the work of supernatural forces? Maybe the person was cursed with it.

**W3**: Some people are cursed with it, very well. Blindness could be cursed on someone, being that from the start there was nothing wrong. I know of someone that went to work and was working that morning in the shop and swept the floor all of a sudden they noticed they could not see anymore. There were no symptoms at all, maybe the eyes were doing somehow, and on that spot is how they went blind. What then do we call it? It’s the work of supernatural forces.

**I**: Thank you ma. Do you have any other explanations that you think could cause blindness asides what you have said?

**W3**: Ehn, another thing that could cause it is hypertension, when it has been in the body for long and affected the body severely it usually causes it. But some can be rectified if it’s monitored early. Some if not monitored early may not be rectified because when they get to the hospital and they see the person has hypertension until when the hypertension is cured before they find what they can do for the eyes. If the hypertension is not cured, they would not know what’s wrong with the eyes. So hypertension too causes it many of the times. That’s what I have to say.

**I**: Thank you Woman 3. Woman 4, I want to ask what your opinion is on inherited blindness.

**W4**: Ah, it’s real. Blindness may be inherited, there’s a family where the grandfather or grandmother had had such eye issues before so that’s the type that could occur with the children. But apart from that, blindness could occur as a result of drug abuse, maybe if a lady is pregnant for example someone who is pregnant unexpectedly, unwanted pregnancy and is looking for a way to terminate the pregnancy. Maybe as a result of the horrible drugs they’ve taken, maybe that’s what has caused for the child to be blind. Then if there’s a couple and the man is violent and usually beats his wife, it’s dangerous to beat a pregnant woman that too could cause blindness or deafness.

**I**: Thank you Woman 4. Woman 5 I want to ask what your opinion on inherited blindness is.

**W5**: What I know about inherited blindness. If a person has em… for a family that I know, that blindness is in one of the parents. So they also inherited it from their parents such that they are five children and four of them are blind. So it’s just one who is not blind amongst them. You know something like that is hereditary. So like that. There are also some that didn’t inherit it, maybe God wanted it so. Because there is a child in this school, they said that’s how he was born, that he was born blind and it occurred in the bible too.

**I**: Thank you Woman 5 for your explanation. Woman 1, I am coming back to you. I want to ask what your opinion is on giving blood sample for a research.

**W1**: My opinion? It’s good, it’s good for one to give blood sample for a research. It’s good that one give blood sample for a research because one would not know the kind of disease in their body. If one leaves a blood sample so that they would know what it is that person’s body. So, it’s good that one gives blood sample for a research.

**I**: Thank you. Woman 2, what is your opinion of leaving blood sample for a research?

**W2**: Giving blood sample for a research is something that is important and very good. It will help someone know the path they would follow if the disease is in their body so they would monitor it and it won’t propagate in the body. So, they would finish it from the onset. So, the children that they may have, they will tell them in time that when they are at some age or at some point in time they should take a test and monitor themselves because in the family, in their blood there is a certain disease. So it’s very good.

**I**: Thank you Woman 2. Woman 3, I want to ask that this research I’m talking about, maybe this research we are conducting at the moment, we then tell you that we need blood samples. We want to know what causes blindness in the blood. What is your opinion about leaving blood sample for something like that?

**W3**: It’s for a cure, there’s nothing one would do with it. It’s to know the solution towards it, there’s no problem.

**I**: Thank you ma. Woman 4, I want to ask you, does your opinion differ from what these women have said?

**W4**: My opinion is the same.

**I**: Okay. I then want to ask you what your opinion is on collecting the results of the blood test. After the research has been done would you like to receive the results?

**W4**: Ah, it should be collected. If one would not collect the result then there is no need to leave a blood sample. Anyone that leaves a blood sample is mandated to collect the result of the test so that one would know the solution to the problem. And if it is good, there is no problem.

**I**: Thank you Woman 4. Woman 5, I want to ask if your opinion is different from what the other women have said.

**W5**: It’s not different, it’s the same.

**I**: So you would like to collect the result of the test on your blood sample?

**W5**: Yes oh.

**I**: So we all agree or is there any different opinion?

**Chorus response**: There is no difference

**I**: No difference?

**Woman in background**: Yes

**I**: Thank you so much. Woman 1, I want to ask that for a research, if you are asked to leave blood sample, saliva or faeces. Which would you prefer to leave behind among the three?

**W1**: Between saliva or blood?

**I**: Saliva, blood or faeces? Which would you prefer to leave behind among the three?

**W1**: Which will be convenient – **Interviewer interrupts**

**I**: Which will you **prefer** to leave behind?

**W1**: For I myself?

**I**: Yes, you yourself.

**W1**: I would prefer saliva

**I**: Saliva is what you would prefer?

**W1**: Yes

**I**: Okay thank you. Woman 2, which would you prefer to leave behind?

**W2**: Blood

**I**: Blood is what you would prefer?

**W2**: Blood is what I would prefer to leave behind.

**I**: Why would you prefer to leave a blood sample?

**W2**: You know that blood is what sustains the entire body. Through blood can we live. So it’s blood , it’ll quickly enable them to find what the problem is.

**I**: Woman 1 let me get back to you. What is the reason for preferring to leave a sample of saliva?

**W1**: Because the saliva is most convenient, it is not easy for someone to be pricked with a needle. It’s not easy so it’s saliva that’s most convenient for me.

**I**: Thank you Woman 1. Woman 3, which would you prefer to leave behind among the three?

**W3**: **Laughs softly**

**I**: Saliva, blood or faeces?

**W3**: **Laughs softly** I don’t know which one I would give because…

**I**: What is the reason ma?

**W3**: Anyone that is – **Interviewer interrupts**

**I**: Which one would be most convenient for you, which if we ask for any of the three which would you readily give among the three?

**W3**: Blood is convenient, saliva is convenient it’s the faeces that’s not convenient.

**I**: Which one? Choose one from the three. And you will state the reason for choosing that one.

**W3**: Mm I can’t choose – the saliva is okay.

**I**: Why did you choose saliva?

**W3**: No reason **laughs softly** It is easily available in my mouth.

**I**: Woman 4, I want to ask which is most convenient or which would you prefer among these?

**W4**: **Laughing softly** I would prefer to leave a sample of blood.

**I**: What is the reason ma?

**W4**: The reason is that it is in the blood that you’ll find the original source of what is happening. It will be found in the blood. That is why I would prefer to leave a blood sample.

**I**: Thank you. Woman 5, which would be convenient for you to leave behind among the three?

**W5**: There is none that I can’t leave behind. If it is for my wellbeing there is none I cannot leave behind.

**I**: Thank you.

**Woman asks question in the background**

**I**: You can ask whatever questions when we finish. Woman 6 is just joining us so…

**Woman in background** Let her be on one side.

**I**: Okay, Woman 6 which would be more convenient for you to leave behind for a research between saliva, faeces or blood?

**W6**: Saliva

**I**: What is the reason ma?

**W6**: Because it is easy to be spat out

**I**: Okay ma, thank you ma. Woman 1 I want to ask you that is there a culture or belief that has a negative view on leaving a blood sample in the society you reside?

**W1**: Ah, eh… in some places there is such culture and in others there isn’t. But in my area there isn’t such culture that prevents one from leaving anything behind.

**I**: Okay, thank you. Woman 2, is there a culture or belief that is against leaving blood sample in your society?

**W2**: In my society of residence, there isn’t. In my village there is no such culture that inhibits one from leaving a blood sample for test.

**I**: Thank you Woman 2. Woman 3, is there a belief or a culture that is against leaving blood sample in your society or your village?

**W3**: There isn’t.

**I**: There isn’t. Thank you. Woman 4, I want to ask if there is a culture or belief in your society of residence that is against leaving blood sample for research or something else.

**W4**: There isn’t.

**I**: Thank you. Woman 5 is there a culture or a belief that is against leaving a blood sample in your society?

**W5**: There isn’t.

**I**: There isn’t. Woman 6 is there a culture or belief that is against leaving a blood sample in the society where you reside?

**W6**: There isn’t.

**I**: There isn’t. I want to ask you all, Woman 1 if for the research we ask that you leave your blood sample but you won’t benefit from the research immediately. Would you still agree to participate in the research?

**W1**: You mean for me? If it is that I won’t benefit from it immediately

**I**: That you won’t benefit from it immediately. Would you still agree to leave your blood sample?

**W1**: So you would now take my blood away for free? **Soft background laughter**

**I**: What I’m asking is that during the research we may find something that would not be beneficial to you immediately maybe for someone else. Would you still be willing to leave a sample of your blood to help others?

**W1**: Mmmm the thing is I would be willing but you and I would have the agreement. That’s what it is.

**I**: What kind of agreement are you talking about? **Woman says money in the background**

**Women laughing in the background**

**I**: Please let us be together.

**W1**: Ehn, I can leave it behind.

**I**: You can leave it behind but what agreement were you talking about?

**W1**: Agreement on money. That if the results come through and it does not favour me I’ll still have something to benefit. You just can’t take my blood just like that. So that’s the agreement we would have.

**I**: Thank you ma. Woman 2 would you be willing to leave your blood sample for research even when you might not benefit from it?

**W2**: **Talks in a low tone** I can leave it behind.

**I**: What did you say ma?

**W2**: I can leave it behind

**I**: Thank you. Woman 3 would you be willing to leave a blood sample for research even when you might not benefit from it?

**W3**: There are different types of blood, Uncle.

**I**: Okay…

**W3**: If they want to take my blood sample would they use a needle to take a tiny bit?

**I**: Yes

**W3**: To know what is wrong with someone. But to take a large amount of blood, I can’t give a large amount of blood. **Women laugh**

**I**: No the blood we are talking about is only about a tablespoon

**W3**: Ah, no no. **Women protesting in the background about the quantity**

**I**: A teaspoon.

**W3**: That you will test to know what’s going on.

**I**: For a test like this it’ll be about a teaspoon, enough to fill a syringe.

**W3**: I can’t leave a large amount of blood sample. **laughs**

**I**: Is it because you would not benefit from it? Or…

**W3**: Not that, you know some people when blood is drawn from their body they will be faint. So I don’t know if I will be faint or not.

**I**: The quantity of blood will not make you faint because I collect blood well. You have up to 4 or 5 litres of blood in your body. So a syringe cannot make you faint.

**W3**: If it won’t affect me there is no problem.

**I**: Thank you ma. Woman 4, would you agree to leave a sample of blood even if the research may not benefit you?

**W4**: For free, there is no problem.

**I**: What did you say ma?

**W4**: Salvation is free, there is no problem.

**I**: Okay. Thank you ma. Woman 5, is your opinion different from the other four?

**W5**: It’s not different.

**I**: So you will leave your blood sample?

**W5**: Yes

**I**: Thank you ma. Woman 6, would you agree to leave you blood sample?

**W6**: I can leave it behind but I would look for something to replace it.

**I**: Like what ma?

**W6**: The blood that is there has been – as I have used it, it has…

**I**: I am listening ma.

**W6**: The blood that is there has been – you know, it has – there is no way you take something there that one would not know.

**I**: So what do you think one could do?

**W6**: What I would do to replace the – **Background chatter and laughs**

**I**: Okay, thank you ma. I want to ask what your opinion on treating hereditary diseases is. Woman 1.

**W1**: It’s good. It’s good to treat it because if it is treated on time it would not affect other relatives, other generations to come. So it is very good for someone to treat it.

**I**: Woman 2, do you think there are treatments for such diseases?

**W2**: With all the various science that is present today, I believe that it has a cure.

**I**: Woman 3, do you have a contrary opinion to Woman 2 that heritable diseases have a cure?

**W3**: I don’t have a contrary opinion, if they conduct a research and there is something that can be done, it is good to do it so it doesn’t multiply.

**I**: So you have the opinion that it is treatable?

**W3**: If there is a cure available.

**I**: Okay thank you ma. Woman 4, do you think that you could prevent hereditary diseases of the blood?

**W4**: It is possible.

**I**: How do you think we could prevent it?

**W4**: Maybe we could prevent it in terms of research and the science of today. So it is possible to prevent it.

**I**: Thank you Woman 4. Woman 5 what ways do you think we could take to prevent hereditary diseases of the blood?

**W5**: Hmm the way I know is in terms of proper treatment. If blood samples are taken like you had said previously, and it is tested and drugs are administered to people appropriately it could cure if taken properly.

**I**: Thank you Woman 5. Woman 6, do you think that hereditary diseases are curable?

**W6**: It’s curable.

**I**: What ways do you think we can cure it?

**W6**: If one goes to the hospital, whatever is prescribed, if one should take it religiously. **Background interruption** … I didn’t inherit any disease, I’m the only one in my house. **Background interruption continues**

**Clamouring to end the interview**

**I**: I want to ask Woman 1. What is your opinion – **Background interruption** It won’t be long before we finish. Woman 1 what is your opinion about using the questions we ask during the research to tell some other people who are also conducting a research in order to assist them. What is your opinion? Would you be okay with it?

**W1**: For you to ask me questions?

**I**: The questions I have asked and the results of the blood samples would you be okay if we used it to assist others conducting similar research. Would you be okay with it?

**W1**: I would be okay

**I**: Thank you. Woman 2, would you be okay with such?

**W2**: I would be okay.

**I**: Thank you. Woman 3 I want to ask that asides that, you know some research where we take your blood samples but some things might be beneficial. Would you agree for us to share that with someone else?

**W3**: What could be beneficial?

**I**: Okay like if we take your blood sample and conducted the research, we saw that we could cure some ailments in it. Some others conducting their research if we tell them and they make a drug for it and gave it to people. Would you agree to such research?

**W3**: It okay like that.

**I**: Woman 4, how about you?

**W4**: There is no problem, it is okay like that. If it would be of benefit to others there is no problem.

**I**: Thank you ma. Woman 5, is your opinion different to what has been said?

**W5**: It’s not different

**I**: Thank you ma. Woman 6, is your opinion different to what has been said? Maybe you are against using your samples or answers to assist others conducting a research.

**W6**: It’s not different, it’s okay.

**I**: Thank you ma. We will conclude with this question. I want to ask you Woman 1, if we want to conduct a research like this in your society. What would be the explanations you would like to hear for you to participate in the research? What explanations would you like to hear?

**W1**: These ones you are giving to us. It is okay like that

**I**: Thank you ma. Woman 2, what kind of explanations do you think we would have to give you or people in your society for them to participate in the research and give blood samples to find out what is in it?

**W2**: Please come again.

**I**: What I am saying is; if we want to conduct a research that involves taking blood samples in order to find solutions to blood diseases maybe you or people in your society. What sort of explanation would you like or do you think we would give them that will make them willing to participate in such research?

**W2**: The explanation you would give them is not more than what you have given us. So that in a way that they will understand properly, that will have meaning to them. That’s what I think can happen.

**I**: I don’t know if anyone has any other explanations in mind that we could do.

**Women Chorus answer** The same method of delivery you have given us.

**I**: Okay Woman 4, I am listening.

**W4**: Concerning how you – you know, your delivery is important. So as you have delivered the speech to us when you get to the community if you deliver it to them in the same way, they will be willing to participate.

**I**: Thank you Woman 4. Does anyone have something to say?

Okay I am listening to you Woman 3.

**W3**: What I have to say is how you deliver it saying “the blood sample, if we do this, if we do that, this is how it will be”. One would say the importance and the benefits in it. So whoever wants to participate, if they are willing they would have it in mind that if I do this for the future and they will do it.

**I**: Thank you Woman 3. Is there anyone else with a different explanation or a contribution to all that has been said from the start?

**Woman complains of time** There is nothing to add and nothing to remove; it is okay the way it is.

**I**: Thank you Woman 1.

**I**: Woman 2, do you have anything to add or an opinion?

**W2**: There isn’t.

**I**: Woman 3 how about you?

**W3**: There isn’t.

**I**: Woman 4 how about you?

**W4**: Nothing to add, nothing to subtract.

**I**: Okay ma. Woman 5 how about you?

**W5**: Yes, there is nothing to add.

**I**: Woman 6?

**W6**: Nothing to add

**I**: Thank you very much. Before we leave I want to ask Woman 1, how old are you? And what work do you do? Where do you stay? What is your level of education?

**Woman 1 laughs softly** I am not old, 24 years old. **Background chatter**

**I**: What is your level of education? Okay, thank you for your explanations. I am very grateful. God will grant us wellbeing and we will see later. Thank you very much.

**Woman in background** Can we ask questions now?

**I**: You can ask questions now.
