# Supplementary material for: The social construction of genomics and genetic analysis in ocular diseases in Ibadan, South-western Nigeria
Source: PLoS One. 2022 Dec 1;17(12):e0278286. doi: 10.1371/journal.pone.0278286 (PMC9714877; doi:10.1371/journal.pone.0278286)
Supplement: S1 Appendix — (ZIP) [file pone.0278286.s001.zip › FGD Guide on Genomics research and Ocular Diseases in Nigeria.docx]

FOCUS GROUP DISCUSSION GUIDE ON THE KNOWLEDGE, ATTITUDE AND PERCEPTION OF GENOMICS RESEARCH AND INHERITED OCULAR DISEASES

1. Please give a brief description of your: age, gender, educational level, occupation, income and residence.
2. What do you know about genetics and diseases?
3. Are there diseases that are inherited?
   1. Please mention a few inherited diseases that you know?
4. Are there any words, phrases or idioms in your local language that can be used to describe genetics/genomics?
5. Do you know of any blind person in your community?
   1. Probe for their age, and ask if they know of any young person with blindness?
   2. Probe for what they think could be the cause of their blindness?
6. What are some of the causes of blindness that you know?
   1. Probe about other beliefs such as spiritual, or other reasons for blindness
   2. Probe to understand their perception and heritability of blindness
7. What are your views about blood donation or taking of blood for the purpose of research?
   1. Probe to find out their views on receiving the blood test results
8. Which body fluid will you prefer to give for research: blood, saliva, or stool and why do you prefer what your choice?
9. What culture and religious beliefs about blood which exist in this community?
10. Probe to know how strong such beliefs in the life of the people in the community
11. What is your view about taking blood for the purpose of research to know the genetic diseases that a person may have? (discuss the relevance of genetic testing)
    1. Would you readily give blood for blood tests /genomic research to detect inherited diseases generally?
    2. What are your thoughts about knowing the results of the test ahead of disease onset?
       1. Probe to know if they would want to know the results of genomic tests performed on them? Give examples: If a test is conducted for blindness and they have the gene for blindness. Would they want to know this results even though currently they do not have any problems?
    3. Would you want your results to be identifiable to others or you want your results to be for you only?
12. What are your views about a research where the participant may not be the immediate beneficiary of the research?
13. Would you be willing to be part of a research that the benefit may not be immediate, and are you willing to participate in genomic research?
14. Would you be willing to take part in a research for the benefit of others?
15. What are your views about relevance of Genomics Tests in Nigeria?
    1. Do you think it can be relevant to the community?
16. What do you think needs to be done to conduct a genomic research in this community?
    1. Probe to know the possible challenges one can encounter in conducting genomics research in this community
    2. What do you think the possible solutions are?
    3. In this community do you think people will be willing to participate in genomic research
    4. Are you willing to participate in genomic research?
17. From whom should permission be obtained before performing genomic research in this community?
18. What is your view and what can be done about treatment of inherited diseases?
    1. Probe to know their thoughts about whether it can be cured?
    2. Probe to know their thoughts about whether it can be prevented?
19. What do you think of sharing your data (genomic results) with a third party?
    1. Probe to know their thoughts and preferences
    2. Some researchers use the data for other useful purposes apart from the study. What do you think of using data for other purposes apart from the study?
20. Before a research/genetic research is carried out what information would you want to be given?
21. Is there any other information that you want to provide for me about genetic diseases/inherited diseases/genomic research?

Thank you for your time. It is greatly appreciated
