# Supplementary material for: The social construction of genomics and genetic analysis in ocular diseases in Ibadan, South-western Nigeria
Source: PLoS One. 2022 Dec 1;17(12):e0278286. doi: 10.1371/journal.pone.0278286 (PMC9714877; doi:10.1371/journal.pone.0278286)
Supplement: S1 Appendix — (ZIP) [file pone.0278286.s001.zip › IDI 01 Female.docx]

I: Ok so, your age, your gender and your residence.

R: Ok, I am 46 years old. I am a female. I live at XXX area, Ibadan.

I: Alright. Thank you. That’s all. So do you know anything about genetics and diseases? Silence…

R: Hmmmmm, ah, what I know about diseases?

I: And genetics.

R: I don’t know anything about genetics.

I: Genetics but you know diseases?

R: Mmmmm.

I: Okay. It’s okay. So what are diseases?

R: Ahmmm, like, like ehh, toilet disease?

I: Ehhh.

R: Yes.

I: So do you think that there are diseases that are inherited, that diseases can be inherited?

R: Ehmm, there are many diseases that can be inherited.

I: Interjects, that can be inherited. Okay can you mention like about two of them?

R: Hmmm…Ah! Laughs….

I: Smiles…what are the diseases that you think can be inherited, can you mention two of them?

R: Like AIDS now.

I: Can be inherited?

R: Yes. AIDS can be inherited.

I: Huuu. Okay. Do you know any blind person?

R: I know many.

I: But do you know that blindness can be inherited? Do you know?

R: Yes. It can.

I: Blindness can be inherited?

R: Yes, it can be inherited.

I: Okay. So you said you know blind people, you know blind people now…

R: Yes, I know them now.

I: But do you know anything that might have caused blindness in people that you know? Do you have anything, know anything that might have caused blindness in them?

R: Erhmmm, like if the child is not being taken good care…

I: Interjects, okay the person might get blind?

R: Yesssss.

I: Okay. So is there another thing that you think might cause blindness. Some of your friends that are blind, you have some friends that are blind?

R: Ehhmm…

I: Some of them do you know what caused their own blindness?

R: I don’t know oooo.

I: Okay you don’t know?

R: I don’t know ooo. Some can be caused by the, glaucoma

I: Okay, glaucoma?

R: Ehh,. Glaucoma.

I: Okay, okay, okay. So what are your views about blood donation or taking blood sample for the purpose of research?

R: Ahhh. Ehmmm, that blood donation, I have never doing it, I have never done before.

I: Okay.’

R: Shee you understand?

I: Yes.

R: But people use to do it and I do not discourage them.

I: Okay. So you do not have any issue with for, for, about it. Maybe I come to take your blood tomorrow to do some research about your blood (R: Ahhh), Do you feel to be part of the…

R: No, no, no, no.

I: Okay, you wouldn’t want to be part…

R: I wouldn’t want to be part of it, yes.

I: So, why?

R: Ahhh! I cannot even explain but I don’t have, I don’t like it.

I: Okay. So which body fluid would you prefer to give, is it your saliva, is it your blood or is it your stool?

R: That? That one is not clear to me.

I: Okay as we are…okay you said you wouldn’t want to give your blood?

R: Yes.

I: Now, which other body fluid can you give? Can you give saliva, or stool…(R: Ahhh!), since you cannot give…

R started laughing…

I: Nooo naaa, I want to know, laughs along with her…

R: How will I give my, how will I give my saliva?

I: Ah! I want to do research na so I need your or your kini…

R: Ahhhh.

I: You know some people, during the research we find if there is any gene that causes blindness in you or any gene that runs in your family or so. And I can only do that by getting a fluid from your body. If we want to get a fluid from your body, which one can you give me? Is it your blood, is it your saliva, is it your stool? I want to shaa test and know. Which one can you…

R: Okay. Maybe I can give you my saliva.

I: Your saliva?

R: Laughs….

I: Laughs….why naa, why? Why do you want to give me saliva? They both continued to laugh…why do you want to give me saliva?

R: Shebi you asked me to, to choose my choice?

I: Laughs…ehee naa.

R: Eheee, my choice is saliva. Laughs…

I: So you prefer saliva?

R: I prefer that one than blood. Laughs…

I: Ah. Okay ooo. Is it because you don’t want us to shook you needle, abi what? But you shaaa…

R: Hmmm

I: You just prefer saliva to…

R: Hmmmhu.

I: Okay. So are there culture and religious beliefs about blood in this community? Is there anybody….belief about blood? Any kind of belief about blood in this community?

R: In this comm….ehmmm….hisses…

I: You don’t think so?

R: I don’t think so. I don’t think so. I don’t think so.

I: So but I want toknow your view about, just like I have explained earlier. About taking blood for the purpose of research to know genetic disease that a person may have, you don’t support that?

R: Hmmm. Okay…Though it is…

I: I want to know, you know, you don’t suppose to…I just want to know if you can support it, you will tell me. Do you understand, I’m not like forcing something, I just want to…your idea.

R: Because it is at least it is only through the blood that someone can know the, what is actually going on…

I: What is going on in the family…okay. But research, you will not want to give your own blood for that kind of thing to be done…

R: Hmmmm but if it is, if it is important to you to do…

I: Yes…

R: Ehhhe I can do it. I can release it.

I: Okay. But what is your view about a research where the participant, hmmm, may not be the immediate beneficially of that research. Like I come to you and I take your blood sample to come and do research that will, you know, benefit everybody in your family….let me come and do…at least it is going to take five years before the result will come out. Maybe five years the person may not be around or the person may be around or the person may not the immediate person to benefit from that research that the person donated blood for, so what is your view of about it, will you still participate if you are the person, will you still participate or you will say no ooo…

R: Since it is for my family, I can participate.

I: Okay. Yes, okay. Ahmmm, what are your views about the relevance of genomics, genomic test in Nigeria, genomic tests are test that involve the gene. What are your view about the relevance, do you think it is relevant in this Nigeria, this our country? Doing genetic test, do you think it is relevant? Not by….you understand what I mean? Just like sometime in the past, erhmmm, this type of genetic test was done…

R: Yes.

I: That made us know that if AS marry AS, they will give birth to SS…

R: Interjects with yes, yes, yes, I even did it when I wanted to marry.

I: To marry abi?

R: Yes na.

I: Shee you understand now. I want to know, do you think that, what is your view about that.

R: It is normal. It is good.

I: It is relevant in Nigeria?

R: Yes. It is relevant, yes.

I: And you think it can be relevant to this community as well. Do you think it can be relevant to this community, maybe we want to test and know? Like is there anything in anybody that is causing blindness in this place. Do you understand?

R: Yes.

I: Ehh, do you think it can be relevant to this community?

R: Yes, it can.

I: So what do you think needs to be done if we want to come and conduct such research in this community? What do you think should be done?

R: Ehhh, you know, firstly, firstly come, and involve ehmm, educate people.

I: Okay, okay.

R: About what you want to do. Shee you are understanding me?

I: Alright.

R: So by doing that, so it will make, make it easy for them, shee you can understand me? To….

I: Participate

R: To participate.

I: Do you think if we tell them they will be willing to participate, do you think people of this community will be willing to participate if we explain to them?

R: If you explain to them, they will, they will, they will.

I: Okay. So ehmmm, from who should we take permission from, before conducting this research in this community, is there any body that we need to take permission from?

R: You know I do not belong to this community.

I: Okay. That’s true.

R: This is ehmm, Adeshola area. You know I told you I came from OLorunshogo…?

I: Yes…

R: Hmmm, but, but if you want to do such a thing, maybe you ask for the landlord, landlord chairman.

I: Okay…

R: Shee you understand me?

I: Of this community.

R: Of this community. So when you go to him and explain, he is the one to help you, shee you understand me?

I: Okay

R: To help you communicate to others.

I: Communicate to others, yes.

R: Of what you want to do at the program, soo…

I: Okay. What is your view about the treatment program for inherited diseases, do you think, diseases people inherited, do you think they can be cured?

R: Ahhh. I don’t…

I: Do you think they can be cured?

R: I don’t think it can be cured. It cannot be cured ooo. Inherited disease.

I: But do you think they can be prevented?

R: Yes! They can only prevent it.

I: And it is by finding out if there is any disease someone inherit in the family ooo, it is by this research now. If I do a research now and find out that, ah, this family has this, this, bla, bla, bla, they have this thing ooo, they will start, you know, working on it, let me just say, someone’s mother died of cancer, now we are trying to run, ehmm, a genetic test in that family to know whether there is a gene causing cancer in that family. If I know, at least everything that concerns that person, I will find every kind of treatment or preventive measure I need to take on that person on time..

R: Yes.

I: Before that kind of a thing will happen to…

R: Yes, yes.

I: Do you understand, so that is the reason behind this…can be prevented.

R: Not cured.

I: So what do you think of sharing your idea with a third party? Like if I take your, your, about sharing your…rather. If I take your blood now to go and do some test and you know I will just store it in wherever we store our blood and so someone wants to come and do a research on this community, and the person will now come to me, shebi, you’ve done research in this community, please give me their blood ooo let me use, what do you think about that, do you like it?

R:Ahh! I wouldn’t like such.

I: You won’t like such abi?

R: Ahh, Yes.

I: Okay. It’s okay.

R: hmmmm.
I: So what are your thoughts about knowing the result of your genoms, like would you like to know the result of your test if we perform any…

R: If you…yes naaa.

I: So would you like to know the information ahead the disease onset or you will not prefer to know at all?

R: I would prefer to know it.

I: Okay. So would you like your own to be identifiable to others or you want your own to be for you and for you alone? Do you want others to know about it?

R: Ahh! Noooo. I wouldn’t want others to know.

I: Okaty.

R: Yes.

I: So before you…what information would you like to be..if the..whay information would you like me to give to you? Before I can finally..

R: ehmmm, at least you will tell me what needs to..

I: What I need to do

R: What I need to do. What…yes.

I: Okay is there any other information that you want me, you want to provide for me about genetic diseases or inherited diseases, is there any information that you would want to tell me? Any other thing that you want to tell?

R: I don’t have much.
I: Okay.

R laughs….

I: Thank you for your time, I really appreciate you ma.

R: Laughs…thank God.

I: Thank you so much.

R: Thank God.
