# Supplementary material for: The social construction of genomics and genetic analysis in ocular diseases in Ibadan, South-western Nigeria
Source: PLoS One. 2022 Dec 1;17(12):e0278286. doi: 10.1371/journal.pone.0278286 (PMC9714877; doi:10.1371/journal.pone.0278286)
Supplement: S1 Appendix — (ZIP) [file pone.0278286.s001.zip › IDI 02 Male.docx]

IDI 2 (KAP).

I: Can you tell us your age, gender and residence

R: I am male, am now 39

I: Ok.

R: I live at XXX area, Ibadan. I’m a male.

I: Ok. So I want to ask you, do you know about genetics and diseases? Genetics and diseases, do you know anything about them?

R: Yes. There is disease and there is genetic.

I: But do you know, can you tell me few things that you know about genetics?

R: Ehhh, we have glaucoma, we have cataract, we have many, many things that cause blindness, yes.

I: Ok, so are there diseases that can be inherited?

R: Yes, it’s hereditary. Some, some are…

I: Interjected, can you mention some of them for me?

R: Some of them are hereditary like..

I: Like….

R: Glaucoma is hereditary.

I: Ok, like heredity, ok.

R: Yes.

I: Ok, that is ok. Ok, so you know ehhmm, a lot of blind people?

R: Yes, I know a lot of blind people. A lot of blind people that have a…

I: Like their ages…?

R: Yes.

I: Like how old?

R: Like they are like forty-five, fifty, that are affected by glaucoma, yes.

I: Like glaucoma, ehhh, so you think that glaucoma is one of the things that caused some of them their blindness?

R: They are even, some of them told me that they were affected by glaucoma, yes.

I: Eheee, so…

R: That is gradual lost of sight and eventually lead to blindness, total blindness, yes.

I: Blindness, ok. So are they some other things that might cause blindness? Like spiritual or any other, you think…

R: Interjected, spiritual, spiritual things cause it..

I: Spiritual can cause it, ok.

R: Yes. Like my own is spiritual cause, yes.

I: Ok. Can blindness be inherited?

R: Blindness can be inherited, actually.

I: Ok, ok. So I want to know your views about taking blood for the purpose of research. Like if I come now and say I want to take your blood to do some research, I want to know your view about it?

R: What I know…noise...

I: I didn’t hear you, what did you say?

R: So taking blood, taking blood is not bad. One thing I know should be done is, the blood should be tested in the laboratory so that whether the person to be given is worthy to take such blood or not so that at the end of the day, it will not result to problem. So it will not cause problem to that person. My view about that is not bad.

I: Ok…

R: Taking blood is not bad. It must be…

I: Done in the lab, carried out by specialist…?

R: Yes, by specialist.

I: So which bodily fluid will you prefer to give for research? Which body fluid will you prefer to give for research? Ehmmm, blood, saliva or stool?

R: Hmmm, blood.

I: Blood you prefer, why please?

R: Ahh, blood is, you know blood is human being.

I: Yes

R: The spirit of human being is blood.

I: Okay.

R: If blood is affected, nothing else again. Human being cannot exist.

I: Hmm, So are there cultural and religious beliefs about blood in this community? Do you know? If there are culture and religious beliefs about blood in this community?

R: In this community, ahh, I don’t know, I don’t know.

I: Ok, you don’t know.

R: I don’t know

I: Ok, that’s ok. So, ehmmm, ok, what did, ok, you just said you don’t have any problem taking your blood for the purpose of research, to know the genetic disease that you may have, you don’t have any problem with that?

R: Hmmmm.

I: So would you readily give your blood for blood test to detect any inherited diseases through your blood test?

R: Hmm, yes, no problem.

I: You will give?

R: Yes.

I: Okay, what are your views about the participant may not be the immediate beneficially of that research?

R: All is well.

I: You don’t have any issue…so you will still give your blood even if you will not be the…

R: Yes, I don’t have, I don’t have any issue, yes.

I: Ok. So what are your views about the relevance of genetic test in Nigeria? Do you think it’s relevant?

R: It’s relevant.

I: Ok, so you feel that it should be continued…?

R: Yes, it should be continued.

I: So do you think it can be relevant in this community?

R: It can, it can, it can be relevant.

I: Ok. So what do you think that needs to be done to conduct a genetic research in this community?

R: Ehmm, there should be public enlightenment

I: Ok.

R: Enlighten people so that people will not see it as taboo or they want to endanger their lives.

I: Ok, so you think if we tell them about hts thing, do you think that the people of this community will willingly, will be willing to participate in the research?

R: Actually, they will.

I: They will if we explain all the processes to them,. Tell them what the research is all about.

R: Exactly.

I: They will be willing to participate?

R: Of course, ofcourse.

I: Is there anybody I should take permission from before performing that kind of test in this community?

R: Pardon..?

I: Is there anybody, who I should take permission from before performing genetic research in this community?

R: Ehh, the health care people. People that are concerned.

I: Ok, we should meet them and…

R: Yes, meet them, yes.

I: And explain our selves.

R: Yes, carry them along.

I: Okay. So what are your views about the treatment of inherited diseases. Like do you think inherited disease can be cured? Do you think it can be prevented? Like what do you think…

R: Actually, it can.

I: It can be what?

R: It can be prevented, it can be cured. Even there is a saying that prevention is better than cure, yes.

I: So you feel inherited diseases can be prevented and you feel they can also be cured?

R: Ah, for instance, HIV/AIDS can be prevented, yes. I mean it can be cured, yes.

I: It can be cured?

R: Yes, it can be cured. It can be prevented. Any one of the two.

I: Any one of the two?

R: Yes.

I: So what do you think of sharing your data with a third party. Assuming I take this data of yours now, I take your blood sample, and then someone wants to come and conduct a research in this community again, and the person now come to me and say, ah, you’ve done this, ehmm, you’ve done a research here before and you still have their data and you still have their blood, please can you give me that instead of me to come here to collect it again, can you give me let me use and do my own research, what do you think about it, do you accept that kind of a thing or you don’t want that kind of a thing or you don’t want your data to be shared or anything?

R: To me there is nothing bad in it.

I: Okay, you don’t have any issue with it?

R: Yes, I don’t have any issues. I don’t have.

I: Okay. So what are your thoughts about knowing the result of your test. Like would you like to know the result of the test performed on you?

R: Yes. I want.

I: Like if a test is conducted for blindness and then we have the gene that causes blindness in you, would you want to know the result, even if you don’t have….

R: Yes, yes, yes.

I: So before the research will be carried out, what information do you want to be given to you?

R: It is left to those who are concern for that research. They will tell me what..

I: Ok..

R: The outcome of the result.

I: What, I mean when I want to come and conduct a research…

R: Ok

I: What information would you want me to give you?

R: Ahh, well, the information that will help me, that will serve as enlightenment to me. That even..

I: Tell you what I’m about to do?

R: Yes, yes…

I: The process, the benefits, the…

R: Yes, yes, exactly.
I: Ok, is there any other information you would like to provide for me about the inherited diseases? Is there any information you want to tell me about inherited diseases? Anything at all?

R: Hmmm, yes, we, you, the information I have, you know, enlighten people. Let people know that such a certain disease, a certain disease can be inherited so people should make sure that they treat themselves and they submit themselves for treatment and medical record to know the, the, their status. So that the disease will not being from one generation to another, yes.

I: Ok. Thank you very much for your time.

R: Thank you. You are welcome.

I: I appreciate.

R: You are welcome.
