# Supplementary material for: The social construction of genomics and genetic analysis in ocular diseases in Ibadan, South-western Nigeria
Source: PLoS One. 2022 Dec 1;17(12):e0278286. doi: 10.1371/journal.pone.0278286 (PMC9714877; doi:10.1371/journal.pone.0278286)
Supplement: S1 Appendix — (ZIP) [file pone.0278286.s001.zip › IDI 03 Male.docx]

IDI WITH AY KAP CONT’D

**I: Interviewer**

**R: Respondent**

I: please help me mention your name sir?

R: My name is XXXX

I: okay, how old are you sir?

R: 41 years

I: Are you a boy or a girl?

R: I am a boy

I: what do you about diseases, and the diseases that are inherited, you know we use to say at times that haa, that kind of diseases is in the blood of my family, like father will have, the child too will have and when the child gives birth, the child will also have, so genetics and diseases, so what can you say about it?

R: hen, it happens like that, it is true, such exist, that the father or mother will have it and it spreads to the children too, so it has been in existence for a long time, since the days of our fore fore fathers it has been there.

I: so is that all you can tell us about it?

R: hen and there is no one it can’t happen to, but if it comes the person should just quickly work on it and take care of herself

I: so do you think there are diseases we can inherit?

R: well, with the way people say it, it may be possible

I: okay, but you don’t know if one can inherit any diseases?

R: huh, bible didn’t tell us we can inherit any disease.

I: ohh, so you are following what is in the bible?

R: yes oo, I am following the words in the bible.

I: okay, so can you mention some diseases, you know one can inherit, may be the ones you have been told before, or you have heard before or the one that you yourself have seen before?

R: hen, the disease that one can inherit, for instance, this my condition that I have now, I am someone that can’t see very well as I am, so if a father or mother can’t see very well to the extent that the disease is now in the blood of the parents, the children can inherit it from there when they give birth.

I: so let’s say you have mentioned “blindness”?

R: yes

I: is there another one that you are aware of that you can mention?

R: hen.. Well, another disease that I know it can be like that is like may be those that don’t hear, may be they can also inherit that one too.

I: do you know anyone who is blind?

R: yes now, after all, I am also blind, I know so many blind people

I: okay, so those people you know that are blind, how old can you say they are?

R: you mean every one of them?

I: yes

R: young people are there and old people, there are some people that are very old and suddenly became blind; it wasn’t that they gave birth to him like that. May be while some of them were working, they have read a lot, graduated before blindness came in, and there are some that have been blind since they were young, so some are born with it

I: so do you even know the why those people you mentioned are blind. Do you know the cause?

R: haa, well you know we just met ourselves there, so you know it is what individuals say, since we are not family, I can’t know maybe that’s what causes their own blindness or not.

I: what are the things you think are the causes of this blindness? Do you know them?

R: well, what I think can be the cause according to what people are saying, is if a woman is pregnant, and didn’t use the drugs she is meant to collect, it can cause blindness for her, then, may be after giving birth to a baby, maybe there is no proper care and also the world ”aye” can also use it to trouble you. For some people who are old, may just sleep and wake up and suddenly realize they can’t see again. So different things can cause blindness

I: so do you believe that world “aye” can cause one to be blind?

R: yes, the world “aye” can cause one to be blind oo

I: so what other thing do you know about blindness apart from the ones we have mentioned earlier?

R: you mean what can cause blindness?

I: yes

R: well, if there is no much care, I think it can cause one to be blind. May be if someone is sick, may be the eyes is paining him and they didn’t take him to the hospital so that he can be treated, he can cause blindness

I: so do you think this blindness can be inherited?

R: yes

I: henhen.. so what’s your knowledge about collecting blood samples when we want to do test? You know there are different types of research centers and when it now relates to someone’s health, if we want to do each test we use to collect some things from someone’s body like blood, urine, feces, saliva, so what can you say you know about collecting blood for test?

R: well, what I can say I know is; for them to know the exact place that is the source of the disease that is happening to the person, may be its in the body system, maybe it was inherited from parents, or there is another place he contacted it, so they collect blood or urine to know the source of the ailment

I: so I also want you tell me you your view about taking blood, so after collecting it for that test, we will now give you the result of the test we did. So what do you know about that? Because there are many people that they will tell they want to do test and after taking their blood, they won’t want to hear the result of the test, so I want to know your view in collecting blood and telling the result.

R: what I am thinking is that, if the person they took blood from is educated and knowledgeable, he should be willing to collect the result of the blood that was collected. Now, that the blood we collected from you shows that, that is why you are sick ooo, or the blood we collected reveal that nothing is wrong with you. Since they have collected ones blood, one must wait to collect the result so that he can know what the cause is.

I: so if they now ask you that which one will you prefer giving between collecting your blood, saliva or feces?

R: hen, if something is wrong with me and they say this is what they want to collect, I don’t have a problem, I will give it, since I am the in that is sick and I want treatment. I will leave it now.

I: which one can you give among all?

R: there is none I cannot give.

I: ok, there is none you cannot give?

R: yes now.

I: do you now think in the neighborhood you live, do you know what culture and religion say about blood?

R: culture and religion?

I: yes, does it say anything g about blood?

R: well, you know individual believe is different.

I: yes, that’s why I am saying where you live or where you come from, ma be your culture says something about blood or your religion says something about blood. These things is what I want to hear from you

R: my religion didn’t say anything about blood whether good or bad. NO no no no

I: what of your culture

R: no oo, I don’t believe my culture says anything about blood

I: okay, so if we now tell you that you should leave your blood to run a test on disease that we inherited or diseases that are in ones generation. You know after taking your blood, we now tell you that the research we want to do is to know may be in your family there is a disease that has been showing since the days of your fore fathers, will you leave your blood?

R: yes, the reason why I will leave it is if I know it is a hospital

I: So baba you said if its hospital, you will leave it right?

R: Yes

I: So what if they told you it’s for research will you agree to leave it?

R: Well, if it’s someone I know I might leave it

I: so what is your knowledge in giving that kind of blood for research, which you will not now benefit from the research?

R: huh, if I won’t benefit from it, ehn, but you know it won’t be useful for me like that so it has no meaning to me like that.

I: you won’t do it? R: yes

I: what if we now say you will benefit from it but not now now now, even if not you will benefit from it, may be your children will be the one to get the benefit.

R: hen, if it’s my children that will benefit from it and I make findings, and it’s true they will benefit from it, I will leave it

I: you will leave it?

R: yes, I will leave it.

I: so what’s now your knowledge about, you know the research study that was inherited in the family or generation that we use to do in Nigeria, what is your knowledge about it? Do you even think that its important to the community you reside or is it even useful at all?

R: its useful hen, you know, the reason why it’s useful is because in those days of our fore fore fathers before enlightenment started, these diseases are what causes for some people that some people when they gave birth, they might say you should knee down 16 times, like 3 or 4 kneeling downs so those diseases are what kills those children those days, but now because of continuous research and more enlightenment, it now result in reduction of those things in our vicinity and community and country.

I: ok, you said you are living here in Adesola?

R: yes, by the special grace of God

I: so if we say there is something we need to do before we start that kind of research in this vicinity, is there someone we need to see before such thing happens?

R: hen, well, I also rented this place, this is not my house anyway but we have chairman in our neighborhood.

I: so someone like that we can go and meet him and ask him

R: yes.

I: so if we want to do that kind of research in your vicinity, what do you think we can do to start such an interview?

R: hen, when you see that chairman, he will be the one to give you a go ahead whether to do it or not?

I: Are there any challenges, we can encounter when doing it?

R: hen the challenges you can encounter is that, you know some people that are not educated may be asking what are they doing, why are they doing it, he may be using idea to ask question about things, so it’s not possible for one not to see any challenges.

I: so what do you think can be the solution to such a problem?

R: what I think what can be a solution is that, hum, this kind of things you are doing now, if this kind of problem is coming up on radio and people are listening to it, and they are enlightening people that they should be doing and someone who has disease should not hide it at home, such an individual should go out, and if some people comes to the neighborhood to do research they should respond to them they should not run away or hide from them that will also enlighten people to know about it.

I: do you now think your people will be happy to partake in this kind of research?

R: yes, they will be happy.

I: what of you, are you ready to partake?

R: haa, if I am not ready, just like as you came to meet me now, I won’t answer you.

I: so what is your knowledge in taking care of sicknesses we inherited?

R: there is nothing, one must take care of it, so that it won’t be…..

I: do you think such kind of sicknesses have a solution?

R: huh, well, because the world is becoming more enlightened, I think it has a solution.

I: do you think we can even prevent it from happening again and again?

R: hen, well, it may not happen again, you know, when you know the cause, and someone can take steps to prevent it from happening again

I: so what do you think we can do about diseases that are inherited?

R: what someone can do is if he realizes he has this inherited disease, he should go to a place they can take care of him so that it won’t be spreading like that

I: so what do you think, that after we are done collecting the information we need from you and we now show someone else that was not where we conducted that interview, like a third party, will you be happy?

R: I will be happy; there is no problem since you didn’t ask me a bad thing and I didn’t answer anything bad, there is no problem,

I: some researchers also use the answers of this kind of research for other things, do you understand?

R: yes,

I: apart from this study. But you know about research very well?

R: yes,

I: so do you think it’s good we use the findings from research for something different from the initial purpose we collected it for?

R: if it’s something that will be beneficiary to the person, that it is something that will not cause harm the person who gave the response, it can be used but if it’s something that can affect the person, no, they should not use it.

I: so, if we now say we will do the research, and we collect sample and the likes from you, will you be happy to hear the findings from the research when we are done analyzing the research?

R: I will be happy

I: let us assume we are done with our analysis and then now realize that the blindness is in your blood i.e. in your generation, will you like to hear that kind of result?

R: yes, why not? I will want to hear

I: or will you like us to give another person that kind of result or you will want to be the only person to hear it?

R: it’s me that you asked that you should give the response to, you don’t need to give someone else again

I: so before we commence this kind of research what do you think we have told you prior the time or which kind of information do you think we tell you before coming for this research? Or is there something we should have told you before that we have not said or what do you just feel?

R: what I just feel is that when you come, you will say you want to find out things like this oo from you and you will tell us where you come from, something like that

I: so is there anything you want to tell us apart from what we have been talking about from beginning?

R: I just have a question

I: ok sir

R: it’s not that you came from a hospital like that. I: yes sir, R: so I want to ask a question, maybe there is someone now, may be a boy or a girl that has this my condition that can see very well, he or she now gave birth, like maybe he has given birth to like two children and nothing is wrong with those children, later, after he gave birth to the third child, something now happen to the child, the child now have this kind of condition, what can you say it’s wrong with that kind of child?

I: that is part of what we are saying, that is why we are doing research about inherited diseases in sight, and do you understand?

R: yes

I: just like you have had it now, your wife didn’t have it,

R: yes,

I: and you have given birth, and now both your wife and children, you will now bring them to the hospital, they will now be taking treatment that will not allow them have it, do you understand?

R: yes

I: so if you also have a child that has it, that is still young, there is a place they can still control it to before it get harder than that. Do you understand?

R: yes

I: that is why they are doing that kind of research, for someone who has it already; he can still be coming for this kind of care so that it can be prevented from getting worse than before. Do you understand? And for someone who has not gotten it at all too, can begin to take treatment that will prevent her from having it at all. Do you understand?

R: yes, but I asked you a question that which hospital did you come from and you are saying a private hospital, you don’t want to tell me

I: thank you for allowing me to record your voice, thank you so much for your time
