# Supplementary material for: The social construction of genomics and genetic analysis in ocular diseases in Ibadan, South-western Nigeria
Source: PLoS One. 2022 Dec 1;17(12):e0278286. doi: 10.1371/journal.pone.0278286 (PMC9714877; doi:10.1371/journal.pone.0278286)
Supplement: S1 Appendix — (ZIP) [file pone.0278286.s001.zip › IDI 04 Male.docx]

IDI with Em 1B-KAP

**I: Interviewer**

**R: Respondent**

I: so good afternoon again, my name is Tunde, please may in know your name, your age, your resident and your gender

R: my mane is XXXX. I am just one year after forty, and I am a man and I live around XXX area of Ibadan.

I: what do you know about genetics and diseases?

R: well, I am not a medical practitioner but genetics to me is something that is related to blood or something that you can get from blood transfusion or transaction or whatever.

I: what of diseases

R: those are the diseases that can affect people and cause damage to one’s body.

I: are there diseases that are inherited?

R: yes, there is oo,

I: like?

R: I think.

I: just mention about one or two

R: I think like diseases that are inherited

I: any diseases, it doesn’t need to be eye related

R: well, I don’t remember the name of those diseases

I: but you know there are diseases that are inherited.

R: yes, they are many disease that are inherited

I: do you know of any blind person?

R: yes

I: not one right?

R: yes, many

I: what are the causes of blindness that you know?

R: causes of blindness, one, heredity is one, conjunctivitis is one, syphilis can do so, accidental, like accident and so on, then injury during birth, then what other thing, misuse of drugs

I: so do you think blindness can be inherited?

R: yes, I am very sure

I: what is your view about blood donation, or taking blood for the purpose of research?

R: yes, once the blood is tested and normal, I think it is okay

I: do you support that, like asking people to donate blood for the purpose of research?

R: for the purpose of research? Hmm, I don’t have idea about that before I could support that, I have to be fully oriented about it

I: ok, if you are fully oriented about that and tell you why the research is been done, can you give your blood for research

R: if I am convinced

I: ok let me rephrase the question, which body fluid would you prefer to give for research; is it your blood, your saliva or your stool?

R: for research?

I: yes

R: because, I am hearing that for the first time,

I: what is that?

R: donating blood or any blood fluid for research, I only use to the one we are asked to do in the hospital for one illness or the other but for research, I don’t have the idea.

I: you know for instance now, there are some drugs that are been invented now, it is as a result of research that they do. For example, the very good example is; do you know about sickle cell anemia?

R; yes

I: you know how they came about people having SS AS, people donating blood whether is compatible or not, that is what people have been doing a lot of time. Sorry let me come back to the question, it seems I am digressing, now the question is even though you say you are hearing it for the first time, I get that, but if like you said if you are oriented about that, if they tell you much about it and explains why they want to do it, what are you comfortable giving out? Is it your blood, your saliva, or your stool?

R: well, I will prefer blood

I: why do you say you prefer blood?

R: because I think, it’s the most common thing that someone can just give and you let go

I: what do you think it’s the cultural or religious belief about blood donation?

R: hen, what I think is peoples belief about blood donation is, once you donate blood, you will be short of blood, so fear about blood are very many some people don’t want to do it.

I: so what is your view about taking blood for the purpose of research to know about the genetic disease that a person may have?

R: haa, well, if for the purpose of research, I think it is ok

I: you know if you are saying you believe blindness can be inherited, anything that needs to genetic has to do with blood. Isn’t it?

R; yes

I: so if you are looking at the relationship or differences you may mix it, now the question is will you readily give blood for blood test or research to detect inherited diseases would you?

R: (smile) if it is in my own case, I can do that.

I: so what are your views about research where the participant may not be the immediate beneficiary of the research? For example, may be they are doing a research now, and the people they are begging that ok, leave your blood when result of the research is out, you may not benefit from it or may be your children or children’s children will be the ones to benefit from it, will you still participate in it?

R: Yes, very well

I: what is your view about genomic test in Nigeria, do you think it is relevant to the community?

R: I don’t think it is relevant to the community, for example now, look at my own case now, I don’t hear it, I dint even know they are doing that for research

I: which means you haven’t heard it and you don’t know the challenges that can be faced, do you know if people will be willing to participate in such research?

R: yes, if they are oriented about it, they can

I: so what is your view about the treatment of inherited diseases, do you think it can be cured?

R: I think it can be cured even though, it may not properly prevented it can be cured at least, those ones that has happened.

I: do you mind sharing your data with a third person, for instance, let’s assume you volunteer to participate in a research and you donated your blood do you mind sharing the result of that blood test with another person?

R: yes, there is no problem,

I: so what are your thought about knowing the result of your test, do you mind knowing the result of your test if you participate in a genetic research?

R: yes

I: you would like to know the result of your test?

R: exactly yes

I: ok, would you like your result to be identifiable to others or you want your result to be for you only, for example, may be we have done the test and research and its many would you like your result to be with the other people’s own or you want it to be yours alone.

R: it should be with other people, there is nothing bad there

I: so before the research is done, like you said earlier, that if someone is properly oriented, he should be able to participate in the research. What is the information you want to be given before you can participate I the research.

R: I will like to know why, what and I will like to know the purpose that’s what I mean by why do you want to do it, then what for, those are the information, then is there any effect or not,

I: is there any other information you would like to provide for me about genetic or inherited diseases?

R: no, I don’t have any Information

I: so Mr Martins, thank you for your time.
