# Supplementary material for: The social construction of genomics and genetic analysis in ocular diseases in Ibadan, South-western Nigeria
Source: PLoS One. 2022 Dec 1;17(12):e0278286. doi: 10.1371/journal.pone.0278286 (PMC9714877; doi:10.1371/journal.pone.0278286)
Supplement: S1 Appendix — (ZIP) [file pone.0278286.s001.zip › IDI 05 Female.docx]

IDI with IY 1A-KAP

**I: Interviewer**

**R: Respondent**

I: we want a brief description of you, how old are you? I brought two different papers. We have finished one, and this is the second one. They want you to talk about, your age, your gender, and where you live, that is what they want you say. That is number one question

R: my age is 45 year, February 10, 1974,

And your gender, are you a boy or a girl?

R: I am a girl

I: and where you leave?

R: XXX area

I: what do you know about genetics and diseases? Genetics is what one can inherit from a father or mother then disease can be malaria, cough, do you understand? So they are asking what do you know about genetics and disease

R: some people can inherit sickle cell through *noise* or fibroid or madness or *(noise*)

I: do you know if there are diseases that one can inherit from a father or mother?

R: yes, that was what I was saying the other time, some inherit sickle cell, madness or typhoid or measles

I: do you know anyone who is blind?

R: yes

I; those people that you know that are blind, how old do you think they are?

R: some became blind at the age of eight, some at the age of 4 or 6

I: do you have idea of the causes of their blindness?

R: yes, that person at the age of eight told me that measles caused it.

I: those other people that you know are blind, what did they tell you caused their blindness

R: one said its measles caused it and another said that was how he was given birth to

I: do you believe in spiritual things, may be blindness can be caused by spiritual powers

R: yes

I: do you think blindness can be inherited from father or mother?

R: yes

I: ok, then about collecting blood. What is your view on blood donation or someone collecting another person’s blood for research, what is your view about blood donation or collecting blood for research

R: God forbid we will not fall sick, it is most likely it is someone’s younger or older siblings that is in the hospital and admitted that needs blood, if the doctors examine someone that is healthy, it is likely that one of the family’s blood corresponds and they will draw like either a bottle of blood that they will buy and add to treat the sick person so that the person can survive.

I: what is your view about someone who did test collecting the result of the test?

R: hen, it’s the owner of the body that knows how its body is, if someone goes for blood test, if they see that the blood is dirty, its either they write the drug that will purify the blood or they inject it.

I: if they want to do test for you for research, which one can you leave? Is it blood or urine, stool or saliva? You know they collect stool for test? So for you now, I am asking you which one will prefer to give, is it blood, urine, stool or saliva?

R: you know, some doctors request for both, either one you give, it is likely that it is through what you eat that you got disease.

I: what I am saying is that, which one can you leave at anytime they ask you? Whether urine, saliva, blood or stool?

R: stool

I: ok, why do you prefer stool?

R: that can quickly show what affected ones blood or the whole body

I: where you are leaving, culture, religion and where you were born and your community, do they have any belief about blood, that when you leave your blood, something bad will happen or they don’t know what they want to use the blood for, and start panicking, or may be belief or culture belief that in this area we don’t leave our blood

R: that is not in my culture

I: what is your view about giving blood for research, and someone to know about disease? Like someone donating blood and them using the blood to know the disease one can inherit either form the father or mother, what is your view about it, like I can’t leave it ooo and nothing will happen to me?

R: *(alati se loun mo bara e se ri), A proverb (its someone that should know what to do to himself)* because there are some people that will have a sickness and will not know it was inherited from his parent so if the person associate himself with those who are well experienced, they will advise him to go for test, and when he gets to the hospital they can request for any one.

I: another one is that, they said, can you leave blood, I mean you, can you leave your blood for a research test to know if there are diseases you can inherit from your parents? If they say we want to come and collect blood and anyone that gives blood we will help them know if they have diseases that they have inherited from father or mother, so I am asking if you can leave your blood for such.

R: me, I don’t believe that, you know the owner of a body is the one that knows the way his or her body is. For me, I don’t observe I inherit anything from my parent either disease or something.

I: what are your views about a research, where the participant may not be the immediate beneficiary of the research? Research sometimes when it is done, you know these interviews we are conducting, it’s a research, sometimes when they are done doing it, they publish it, they won’t write your name they won’t even write anything about you there they will just say some people believe this and some people did not, even those abroad can see it online on internet, and they will come and tell your community leader that the test that was done the other day this is the result, now they are asking what is your view if you are among the research participant and you will not benefit, and they collected all your data now, you will benefit but not immediate, what is your view about that? Like them collecting your blood now for example and you not getting immediate benefit from it, and them not also telling you the result immediately until after a month or two months before you get the result, what is your view about it?

R: hmm me I will be thinking well no matter how is going to be they will still tell me details

I: now, what is your idea about using you for research, but the benefit is not for you but for someone else? Can you partake in such a research, that they will collect sample from you but the benefit is not for you oo but for someone else

R: hen, it doesn’t

I: in this Nigeria, we are, they are asking, what is your view about someone doing test about disease that is inherited. What is your view about them collecting blood, and carrying out a test on the blood for them to know the kind of disease that is inherited by that person, what is your view about it in this county?

R: you know prevention is better than cure, if they do it, it is not bad. Because when I was in secondary school, governors’ use to send doctors, then I was in Plateau that children blind school in Plateau, so they use to send doctors out, if there is any outbreak of diseases, they will send doctors out that will be going round the schools and will be giving us different injections that will cure those diseases, so there nothing bad about it.

I: them collecting blood for test, to know what is wrong with the person, what do you think about it, is it relevant to the community, what they are doing, is not relevant to the community?

R: well, anyone that knows how her body is can wait for that because the body is everything

I: ok now, they now say, if we come to the community where you leave, what do you think one can do for one to be able to collect blood? In the community you reside I mean, so that they can help people search their blood to know what is there that they might have inherited from parents.

R: you mean if they come to my place

I: yes, your place, where you live, you know you are not the only one leaving in the community, what do you think, if we are the ones that wants to come now, you understand, we want you to advice us, what do you think new can do that will make people to release their blood, so that we can run the test to determine if they have this, or inherit that. What do you think we can do? What are the challenges you think we can encounter? What are the possible solutions you think to the challenge?

R: you see, that one is very risky, one someone may not believe they want to assist him, he will think they want to draw part of his blood for something else, you know people that are bad have spoilt the good people, so even that can implicate the person you lodge with. That is what I see

I: so what do you think can be the possible solution, how do you think we can go about it?

R: hen, anyone that comes for treatment is who we can do it for, it will not be that you will just enter houses, people will not want to turn up.

I: now, if we come and meet you, I mean you oo, will you be able to participate?

R: since I have known you and can identify you right from the beginning, now, if you want to come they would have known that it is me that you want to meet with, but for someone else to respond they will not concentrate.

I: now, they said, if we want to come to your community, who is the leader you think we can meet.

R: landlord, because, I am not in my permanent site, I am a tenant I rented my apartment, so you can get permission, through landlord or landlady

I: now, they say, about sickness that is inherited, do you think it can cure, or prevent it, or what do you think one can do about the diseases or sicknesses that is inherited?

R: you see, sickness that is inherited from parent is difficult, its only when the creator that is above operate one himself that is when you can be free, if not, I don’t think that kind of sickness can be healed.

I: okay, now assume we conducted a test for you and now and they saw, for example, you have this and that, that you inherited from your parents, can you tell the third party? Assuming I did a test for you, you know we are only two here, and I did the test for you and I gave you the result, can you tell the third party?

R: you see, what I believe is that, from a day old till you clock about twenty years, if there is no bad problem that goes for long, I don’t …

I: some researchers use people’s data, apart from the study they collected it for; they use it for another thing. What do you think about it?

R: like how?

I: I mean if they collect data from people, you know they should publish it so that people can see that this and this is what is wrong with people, so that government can help us to do something about it. So apart from using data for that study, some researchers also uses it for other purpose apart from the study, what is your view about it? Is it good or it is not? Apart from the study they collected it for?

R: hmm.. if they use it for another study, it might annoy some people that they went to put him on internet, that if he know they will put him on internet, he would not have turn up

I: what do you think about someone doing test and testers telling the result to the person, for example, if they do a test for someone about blindness, the person is not blind yet but they did a test and they now realize that this person has a disease called blindness, do you understand and the person is not blind yet. Do you think the person will be happy about the result and that people saw the result?

R: you know there are someone that, because that blindness is of different types, there are some that cause glaucoma, that the eyes will quench entirely, another can cause cataract, that when they do the operation, if its one, the person will see, but glaucoma, even when they do the operation, he can’t see, even if the person is not luck, the bulb in the eyes will met off

I: do you prefer to have information, for instance, this person his eyes is not blind yet so for instance you, they said you should release your blood for test and this thing hasn’t done you but they’ve seen the trace in the blood that this thing will do you o, do you think they should tell you or they shouldn’t tell you?

R: huh! It is because they want to tell me that is why they examine me now, if they say the result, they tell me how to go about it so that they can overcome it.

I: so what we are saying is that, that your result that they give you, should they give you alone, or if they see you are many, should they gather all of you so that they can advice you together or you will want them to tell you your own alone?

R: haaa, once it is the same disease that is still frustrate our lives, if they say it together it is not a problem

I: so what if they want to do research on you, before they start, which information do you think you can give them? If we want to conduct a research with you now, which information do you think you will like to give first?

R: which kind of research is that?

I: any research, it might be research to collect blood or for the blind we want to know what they are facing, how are they coping, how are they faring, which type of information do you think you can give us before we even begin to collect information or blood from you. So that we can know, you don’t want us to do this, you want that. You know, individuals have their rules and regulations, what A wants, B may not want and may also wants, so it depends.

R: well, the information that I can give you is what I said the other time, about how I use to weave things like that.

I: is there are additional information that you want to tell me about sicknesses that can be inherited.

R: the one i know is what I have told you.
