# Supplementary material for: The social construction of genomics and genetic analysis in ocular diseases in Ibadan, South-western Nigeria
Source: PLoS One. 2022 Dec 1;17(12):e0278286. doi: 10.1371/journal.pone.0278286 (PMC9714877; doi:10.1371/journal.pone.0278286)
Supplement: S1 Appendix — (ZIP) [file pone.0278286.s001.zip › IDI 06 Female.docx]

IDI with P6-KAP

**I: Interviewer**

**R: Respondent**

I: do you know about inherited disease?

R: I don’t know much about I only know glaucoma

I: Please tell us your name, age and where you leave

R: XXXX is my name, I am 33 years old, and I live in XXX

I: you said you don’t know much about such disease or what did you say?

R: yes, I don’t know much about diseases that can be inherited

I: but you said you know glaucoma

R: yes

I: so do you know other disease that can be inherited

R: I don’t know

I: ok, apart from you, do you know other people who are blind

R: yes

I: who are they to you?

R: they are my friends

I: your friends?

R: yes

I: like how old are they?

R: they are also mature

I: so do you know what is wrong with their eyes?

R: measles is the cause of some people,

I: what of yours?

R: measles is the cause

I: so you’ve been like this since you were young?

R: yes

I: apart from measles, do you about something else that can cause blindness?

R: some might have cataract, and I know of, well, that cataract is the only one I know

I: do you believe that some things like spiritual can cause eye disease?

R: yes

I: please tell me more about that

R: yes, I believe

I: why do you believe like that?

R: some people when they give birth to them, nothing happens to them but when they see the glory of some children and what they will become in life, they use to get angry of them, that they should see a challenge that will affect them, or it should be another thing, some might be blind, some might be deaf

I: apart from that, do you know of other things that can cause blindness

R: glaucoma can cause it, if anyone that has glaucoma, even it doesn’t matter, it is possible for such an individual to become blind, if glaucoma is the cause of the blindness, among the children that he will give birth too, that thing will generate in them.

I: so what is your view towards blindness?

R: well, in dint think so much about it but you know when things like this come, it has come there is nothing anybody can do about it.

I: but do you believe blindness can be inherited by children from parent

R: if it is not glaucoma, but if it’s glaucoma it can be transferred from generation to generation but if not glaucoma it can’t spread

I: so what do you think about collecting blood for research, like if we come to you that we want to do a research and this research is to know may be the cause of eye defect and we will need to collect blood from you, what will be your view about it?

R: henn.. no problem

I: when they come to you will you allow them to take your blood?

R: yes

I: are you sure?

R: yes

I: so will you like to collect the result of the test you do?

R: yes, I want it like that

I: but if we give you option of something like we want to do test or research to know what is in the blood that causes some diseases but we will need to collect urine, or blood, or stool from you, which one will you prefer us to collect from you?

R I will prefer urine test

I: I mean between urine, saliva, blood and stool?

R: I can leave saliva

I: why not blood or stool?

R: I don’t know that, saliva is what I will be able to leave

I: please explain

R: that is what I can leave, because the blood in me too is not much

I: you said you will leave saliva, why can’t you leave urine?

R: nothing, that’s just what I can leave, that what I wish to leave

I: what of stool?

R: what if I don’t feel like stooling?

I: what if we give you a bottle and say anytime you stool just put it inside and bring it, will you do that?

R: no, I can’t do that

I: are there beliefs in this community

R: no,

I: are you sure?

R: yes, I am sure

I: and then now that I ask you between saliva, urine, stool and blood, you said saliva is what you like to donate, what if we tell you we need blood; will you be able to leave your blood?

R: well, I will leave it

I: are you sure? If we say its blood alone, will you leave it?

R: yes, I will leave it

I: but if we give you saliva as an option, you will leave saliva

R: yes

I: because you said blood is not much in your body

R: yes

I: so if we tell you we want to do a research that will not be of benefit to you but for your generation, will you like to participate in such?

R: yes

I: that we will collect blood for the test but the test or research will not do you any benefit but will benefit some other people in the future, will you want to do it?

R: well, I will leave it

I: why?

R: since it is what you want

I: no it is not a must, what if we explain to you that it is voluntary, you may or may not, will you still leave it?

R: no oo, I won’t give you

I: if I tell you that if you give your blood, it will assist some other people in the future

R: I can’t leave it

I: why do you say that, please explain to me why you will say haa this thing I can’t leave it,

R: if it is something else I can leave it but I can’t leave my blood

I: k, so why don’t you want to leave your blood?

R: because the blood in my body is not also much

I: how do you know the blood in your body is not much?

R: I can feel it in my body

I: so if it is something else we requested for, you will leave it?

R: yes

I: but what if we don’t request for any sample and we just tell you that we want to ask you few questions for the purpose of research that will benefit people in the future, can you do that?

R: yes

I: so what do you think about doing research with blood to test for some diseases in this Nigeria, do you think it is good for us, or there is no need?

R: it is good

I: are you sure it is good?

R: yes

I: so in this neighborhood, is such a research good for them?

R: yes

I: so if we want to do any research that we will collect blood from people in this neighborhood or community, what do you think we are meant to do to enable us do our research successfully for you and other people in the community and we will need to collect blood from them for research, it will not also benefit them but those in the generation to come. So what do you think we can do? What are the challenges that you think we can face? Now you have said you, you can’t leave blood for such thing, so what other challenges do you think we can face?,

R: they also may not be able to leave it, you know I don’t know

I: so what do you think we should do that will help us to have a successful research that will enable people like you to participate in the research

R: maybe you will meet with them, and leave one day off to talk with them,

I: if we are talking with them, what kind of thing should we talk with them about?

I: so what are the things we should tell them that will enable them to cooperate with us?

R: you will talk to them, may be about the kind of research you want to do is what you will enlighten them about

I: but do you think people will be willing to participate?

R: yes, they will see people that will be willing to participate, we will see among hem

I: what do you think we can do for those who are unwilling, okay give us idea, you know this is where you leave, so you are the ones that can advice us, what do you think we can do, please say the truth, just tell me your thought.

R: hemmm… well, may be, you know now, I don’t know how to explain the proverbs, I mean they use to say a small child, I don’t know how they use to say the proverbs, I mean if you give a child something, they use to know signs, I don’t know how to explain it, ok, about talking to people about it and by showing love out may be by given them some incentive. They may participate

I: things like what, please give us idea, things that you know if we give people in this community, they will say like haaa, if you give them this thing there is nothing they won’t be able to do for research.

R: for example now, they just concluded election, you know there are different party, they say ADC gave like 300, PDP, well, it is just the amount PDP gave that was the highest, you know the people that has the highest money are the ones that have higher vote, I am just using that to cite an example, so things like that

I: so if we want to take permission for such research, who are the people we should meet for permission in this community

R: I don’t have idea about that

I: what if we tell you that we want to do a research and we will collect your blood and also that the result will be shared with other researchers, we will show other people and researchers, will you like to do that kind of research?

R: yes,

I: are you certain you will do it?

R: yes

I: I have told you that anything we see in your blood, we will show some other people, will you like to do something like that

R: yes I will like it

I: please tell me the truth, it doesn’t stop you, it doesn’t affect anything, we just want to know how to do such things

R: if I am the only one, I will like to see the result but I won't like it when you show it to someone else

I: so if I will want to show someone what will you prefer us to do?

R: I won’t do it

I: if we tell you that these people we want to show will use it for other purposes that will benefit people in the future, will you change your mind to do it?

R: yes

I: okay then, thank you
