# Supplementary material for: The social construction of genomics and genetic analysis in ocular diseases in Ibadan, South-western Nigeria
Source: PLoS One. 2022 Dec 1;17(12):e0278286. doi: 10.1371/journal.pone.0278286 (PMC9714877; doi:10.1371/journal.pone.0278286)
Supplement: S1 Appendix — (ZIP) [file pone.0278286.s001.zip › IDI 07 Female.docx]

I: Good afternoon once again. My name is XXXX. So I would like to meet you like your age and where you live.

R: My name is XXXX. I’m nineteen years old. I live at XXX Area, Ibadan.

I: Ok. Thank you very much. Please can you briefly share with us how you became blind. How you lost your sight?

R: I lost my sight when I was still seven years old. In year 2007. When I was still in primary two. This happened when I came back from school that day. So it happened maybe just like a minor sickness, just like malaria.

I: Okay…

R: So since then there have been trying to spend money, to go to here and there to look for solution but, they can’t find solution to it and since then I became blind.

Someone interrupted: Just, when you count from one, two, three, four, five, just go to that one before you come back.

I: Okay. Thank you very much. Did you actually, after that one did you, were you noticing it before?

R: No.

I: It just happened that day day?

R: Yes.

I: And since then it became deteriorating?

R: hmmm.

I: Okay. Thank you very much. So what do you think could cause blindness?

R: I didn’t know

I: Okay. But do you temporary think it could be something traditional or maybe…

R: I think so.

I: Ok you think so?

R: Yes.

I: Okay or you think probably it is religion or something?

R: I didn’t know but I think so. I think maybe it is will be traditional something.

I: Ok. So what will you say about blindness, do you think it can inherited?

R: Yes, yes, yes.

I: it can be inherited?

R: Yes.

I: Ok. Base on your experience, do you think this one, your lost of sight is actually inherited?

R: No

I: So why do you think so?

R: The reason is, I didn’t have anybody who is blind in my family.

I: Okay.

R: So I don’t think this one is inherited.

I: okay. Thank you very much. Then I would like to ask…..like the belief now, you said that you probably think that this one is ehhh…being traditional. Something that has to do with maybe juju. Do you think that, what, how strong are you to believe that like what actually prompted you to believe.

R: The reason is after the, after the sickness we try to use some, we try to use some medical attention. We went to UCH, we went to Adeoyo but after series of test, the result will tell us that there is nothing.

I: Okay but when it comes to the issue of blood, what’s your perception about it, what do you think about it when it comes to religious beliefs about blood.

R: I didn’t think it’s blood because anything blood can be transmitted from one person to another.

I: Okay. Then what’s your belief about maybe taking blood for research?

R: Hmmmm, taking blood for research means checking, maybe checking of what happened, what can happen, maybe what happened to a person through his or her blood. So through the research of blood, we can also get what happens to the person.

I: Okay. So if I will ask, what’s your own opinion about blood donation? Have you heard about it before?

R: Yes.

I: so what’s your belief about it?

R: Blood donation saves people’s lives.

I: Okay.

R: Maybe people that have, ehmm, a shortage of blood, through, through blood donation, they can be, they can be saved from premature death.

I: Okay. So then, if I, have you ever done a blood test before?

R: Yes.

I: When was that?

R: Last year, 2018.

I: last year, 2018?

R: Yes.

I: So what’s your view about taking blood and receiving the result? Maybe somebody takes your blood for test and give you the result, what’s your view about it?

R: My view is that I’m okay about it since I didn’t have any bad…

I: Okay. Maybe after doing the test. What’s your opinion about telling a third party of the result?

R: My opinion? I would agree to do the test, but I would be happy if they tell me what they found in the test before sharing with other professionals. At least if they cannot take care of me then let me be aware of the steps I’d take on time in order not to ruin my future and that of my children

I: Yes. About telling a third party about your result.

R: Ehhh, since the result is good, so I think there is no big deal.

I: Ok, what of in a situation where probably this condition or something is actually noticed, whay will be your opinion about telling a third party about it?

R: I won’t be afraid because I know, ehmm, probably there will be drug for it.

I: Okay. So maybe after checking the blood, we tell your mom, or your sister or your own relatives about it?

R: I’m not going to feel bad.

I: Okay. Then I would like to ask, what’s your view about the research. Maybe you are not going to be the immediate beneficiary of the research?

R: I didn’t understand what you are saying ooo?

I: okay for instance now, maybe you know, you talked about the fact that you can actually give your blood for the research

R: yes.

I: eheee, but in a situation where probably, you may not actually benefit from the research immediately, maybe somebody that will benefit from it, what’s your opinion about it?

R: Hmmmm, there is nothing bad in it.

I: Okay, you don’t mind helping others even if it is your own blood, you don’t mind saving other people…

R: Yes.

I: Thank you very much. Then I would like to ask that, I will like to ask that, ehee…noise…

R: please be fast I’m going for…

I: Okay. So even for future generation, you don’t mind giving your blood to save them?

R: Yes.

I: Ok, when it comes to genomic testing in Nigeria, have you heard about it before?

R: No.

I: Ok, genomic testing could be like, maybe people trying to find out the kind of what is in your blood and how it can actually help. Maybe for instance, maybe the cause of blindness, if it can actually be caused through blood and if they can use it to save people’s health, so what’s your view about it? Do you think it is a nice idea?

R: Yes.

I: You think it is a nice, why do you think it’s a nice idea?

R: I think, the reason I think it is a nice idea is that through the, through the checking of the blood, they will know the real, we are going to know the exact problem that the person has so they will know the solution to the problem.

I: Ok. So what do you think is the purpose, do you think people will be willing to do it?

R: Although people will not, people will, people will be against the opinion but I think it is right.

I: So, can you actually encourage people to, maybe, people in this meeting, can you encourage them to do this…

R: yes.

I: Ok. Then what are the possible challenges somebody can actually encounter? Probably now, you said people may not be willing, why do you think people may not be willing?

R: I think people may be, maybe people, people may be feeling that, maybe they can sense something bad through it. through the…of their blood.

I: Okay. Ok. Thank you very much. Then I would like to ask that would you say, ehhe, do you have friends in your community; do they leave you maybe because when you said you were seven when you lost your sight? But your friends there are they still with you or they left you?

R: They are still with me.

I: Okay. Could you please explain your relationship with your friends in the community, how is your relationship with them?

R: Our relationship is ok.

I: Ok.

R: They didn’t, they didn’t leave me because of my condition. And they always support in all the ways, in all the ways I need.

I: Ok now, you said you’ve actually been seeking for cure since that seven years old…

R: Interjected, yes!

I: So what did, so what role did your religion, your education, what role do they play in it? In helping you to be able to get help?

R: Hmmm, I might need maybe a reader for my, for writing my notes or for recording one or two things. They helped me.

I: Okay. Then what about the economic status, how does that help you…

R: They are trying their possible best.

I: let’s say your family has actually been helping you?

R: Yes.

I: Thank you very much. Then…..so how is it easy for you to move around the community?

R: I’m trying.

I: without anybody assisting you?

R: Yes.

I: Okay. Then what about your daily activities, probably when you want to go to school, maybe having your bath and other things?

R: I’m trying ooo. I’m trying.

I: So how easy has it been?

R: I have been doing it myself.

I: Ok. So do you feel your social life has actually suffered because of this challenge…

R: Interjected, no!

I: Of sight, so you still make friends like before or you still do things you like before?

R: Yes, yes.

Background conversations…
