# Supplementary material for: The social construction of genomics and genetic analysis in ocular diseases in Ibadan, South-western Nigeria
Source: PLoS One. 2022 Dec 1;17(12):e0278286. doi: 10.1371/journal.pone.0278286 (PMC9714877; doi:10.1371/journal.pone.0278286)
Supplement: S1 Appendix — (ZIP) [file pone.0278286.s001.zip › IDI 08 Female.docx]

IDI with Tb8-KAP

**I: Interviewer**

**R: Respondent**

I: ok ma, I want to ask you ma, you have said some things but apart from measles that you said can cause blindness, what other thing do you think can cause blindness?

R: I have said it too; I said it can be hereditary from the blood

I: ok, so apart from that, what other thing do you think can cause it?

R: that is the cause; also the world can cause it

I: if someone wants to do that kind of research that I said earlier, who do you think can give us permission

R: if you meet individual and talk with them, they own their body, so you don’t need to collect permission from anybody except its student, you can go and meet their supervisors but if its these working class, they own themselves so they will decide

I: so what is your view about taking care of diseases that can be inherited from the blood?

R: for instance, if one is infected with toilet disease, if the person goes to the hospital, they will recommend drug that the person will use and if the person is using the drugs the sickness will go

I: what I am saying now is the diseases that are hereditary, what is your view about taking care of it?

R: there is nothing about that if it is hereditary; I don’t think there is any special care except that he needs to be prayerful?

I: so do you think it can be prevented?

R: I have not seen the one that can be prevented

I: so the only thing to do is just to be praying

R: at least I have known like three families that inherited it and I didn’t see anything they did to it

I: ok, thank you ma, you know if someone conduct interview with blood to know the diseases present would you give permission to leave this research so that it can benefit some other people without you name appearing there?

R: hen, it is possible,

I: if we want to test your blood, would you be willing to participate?

R: I can do my own

I: and would you be willing to collect it?

R: yes, if they follow normal process

I: what kind of information would you like not to know? You know when someone do a test some will say don’t bother telling me about the result, you as a person, what are the kind of things you won’t like to know?

R: there is nothing I didnt know, is it not my body?

I: yes

R: there is nothing I don’t want to know, as I am I use to go to the hospital, I will go and do check up, and whatever they tell me to do, I will do it, if it is something that can benefit my health, I will just do it.

I: please before research starts, what are the things you would like to know before you give consent for the research?

R: I will tell them the way my condition started, and if I tell them how my condition started, iu will tell them the things I have been using,

I: thank you so much ma, are their other things you can also say about doing a test that will require when it comes to hereditary disease, are there other explanation that you can also give apart from the ones you have mentioned earlier

R: that’s the ones I have mentioned

I: thank you so much, the Lord will continue to keep you and we pray we will meet you some other time
