# Supplementary material for: The social construction of genomics and genetic analysis in ocular diseases in Ibadan, South-western Nigeria
Source: PLoS One. 2022 Dec 1;17(12):e0278286. doi: 10.1371/journal.pone.0278286 (PMC9714877; doi:10.1371/journal.pone.0278286)
Supplement: S1 Appendix — (ZIP) [file pone.0278286.s001.zip › IDI 09 Female.docx]

IDI with Tb9-KAP

**I: Interviewer**

**R: Respondent**

I: Good afternoon once again, my name is XXXX and I will like to meet you, like your age and where you live,

R: my name is XXXX, I am 19 years old. I live at XXX, Ibadan

I: Ok, thank you very much; please can you briefly explain to us how you became blind, how you lost your sight

R: I lost my sight when I was 7 years in year 2007, when I was in primary 2, this happened when I came back from school that day, so it happened just like a minor sickness, just like malaria, so since then we have trying to spend money to go here and there to look for solution but we can’t find solution to it and since then I have been like this

I: ok, thank you very much, so you’ve been able to see before, it just happened that day, and since then it became deteriorating

R: yes

I: so what do you think could cause blindness?

R: I don’t know

I: but do you think it could be something traditional?

R: I think so

I: you think so, or do you also think it could be religion or something?

R: I don’t know, but I think it could be traditional or something

I: ok, then what would you say about blindness, do you think it can be inherited?

R: yes, yes yes

I: it can be inherited? Ok base on your own case do you think your lost of sight is actually inherited?

R: No

I: why do you think so?

R: the reason is because I don’t have any one in my family who is blind; I don’t think this one is inherited

I: thank you very much, then I would like to ask, like you believe now, you said you think this one is somehow traditional you mean like juju

R: yes

I: I want to ask now, how strong are you to believe that, what actually prompt your believe in that?

R: the reason is after the sickness, we try to use some medical attention, we went to UCH, we went to Adeoyo, after series of test, the result will tell us that there is nothing,

I: when it comes to the issue of blood, what is your believe about that, what is your religious belief about blood?

R: I don’t think its blood, because anything blood, will be transmitted from one person to another

I: ok, then what is your believe about picking blood for research

R: hmmm… picking blood for research means checking for what happened to the person through his or her blood, checking through the blood for research, they can also get what happens to the person.

I: I also want to ask, what is your opinion about blood donation? have you heard it before?

R: yes

I: so what’s your believe about it?

R: blood donation saves life, may be people that have shortage of blood through blood donation, they can be saved from premature death

I: then, have you ever donated blood for test before

R: yes,

I: when was that?

R: last year, 2018

I: so what is your view about taking blood, may be if someone take your blood for test and give you the result, what’s your view about it?

R: my view is okay about it, since I dont have any viral *(noise)*

I: may be after doing the test, what is your view about telling the third party about the result?

R: my opinion?

I: yes, about telling the third party about it

R: since the result is good so I think there is no big deal

I: what of a situation whereby a disease or something was noticed, what will be your view about telling the third party about it?

R: I won’t be afraid because I know probably there will be drug for it

I: so may be after getting the result, if we tell your mum, brother or sister about, how will you feel about it?

R: I will feel nothing about it

I: so i will like to ask what is your view about the person that participated in the research and not been the immediate beneficiary of it?

R: I don’t understand what you mean about it?

I: ok, for instance, may be, you know you talked about you giving your blood for research,

R: yes

I: but in a situation, whereby probably you will not benefit from the research directly, may be someone else will be the one to benefit from it, what is your opinion about it?

R: hum.. there is nothing bad in it.

I: so you don’t mind giving your blood, even if it is to benefit other people, you don’t mind?

R: yes

I: thank you very much, then will like to ask that, even for future generation, you don’t mind giving your blood out

R: yes

I: what is your view about genomic testing in Nigeria, have you heard about it before?

R: no

I: ok, genomic testing is just like trying to find out what is in your blood and how it can actually may be cause of blindness, may be it can actually be done through blood and if you can use it to save peoples life, what is your view about it? Do you think it’s a nice idea?

R: yes

I: why do you think, it is a nice idea?

R: the reason I think it’s a nice idea is that through the taking of the blood, they are going to know the actual problem the person has so they will know the solution to the problem

I: so do you think people will be willing to do it?

R: although, people will be against the opinion but i think it is nice

I: so can you encourage people to, for Instance, people in this community, can you encourage them?

R: yes

I: what are the possible challenges, you think people can encounter, probably now, you said people may not be willing, why did you say people will not be willing?

R: people may be feeling that they can say so many bad things, they may say through the (*too much noise*)

I: thank you very much, then I would like to ask, would you say ehn… do you have friends in your community, do they leave you, when you said you were seven when you lost your sight, but your friends then, have they left you or they are still with you

R: yes, they are still with me

I: could you please explain your relationship with your friends in your community

R: our relationship is ok, they didn’t leave me because of my condition, and they are always supporting me in all ways

I: now, you said you have actually been seeking for care since that seven years old that it started, so what role did your religion, education, what role did they play in it in helping you?

R: hmm, they know i might be in need may be a reader for me in writing my notes, for recording one or two things, they helped me,

I: what about economic status how has that helped you?

R: they are trying their possible best

I: so your family has actually been helping you?

R: yes

I: thank you very much, then how is it easy for you to move around the community

R: i am trying

I: without anybody assisting you

R: yes

I: then what of your daily activity, probably when you want to get to school may be having your birth and some other things, so how easy has it been?

R: I have trying my best

I: so how easy has it been?

R: I am trying

I: do you feel your social life as actually suffered because of this challenge?

R: no

I: do you still move around and make friends like before?

R: yes
