# Supplementary material for: The social construction of genomics and genetic analysis in ocular diseases in Ibadan, South-western Nigeria
Source: PLoS One. 2022 Dec 1;17(12):e0278286. doi: 10.1371/journal.pone.0278286 (PMC9714877; doi:10.1371/journal.pone.0278286)
Supplement: S1 Appendix — (ZIP) [file pone.0278286.s001.zip › IDI 10 Male.docx]

**Keys**:

R- Respondent

I-Interviewer

I: Okay, You said you are 50 years old and you are a male, you live in XXX area at XXX, my question for you is what do you know about things you can inherit from parents such as things our parents also inherited from their parents too?

R: You mean diseases?

I: Yes.

R: Before you mentioned disease you know someone can inherit good things from the parents and some people can inherit disease too such as blindness for example if the person is born blind or maybe a woman is born blind and if this woman’s blood is dominant over that of the husband, there is every possibility for them to give birth to blind children. The children will definitely inherit things from their parents. And out of 100% of the things inherited from the parents 97% is inherited from the mother. It is a serious thing before the woman is the one that carried children in cold blood and breast fed the child too. And out of 100% its always tough for a child to pick 3% characteristics from the father.

I: So were you born blind from your mother’s womb?

R: No I was not born blind, I was 5 years when I had measles and I believe my children cant inherit it because I didn’t inherit from my parents.

I: Can you mention other diseases that can be inherited apart from blindness?

R: Yes, apart from blindness, someone can inherit epilepsy, migraine, sickle cell anaemia, there are so many diseases one can inherit from the parents and there is not sickness of men that can’t be inherited. And the inherited ailments sometime cost you more for treatment than something you just acquire naturally.

I: Do you mean it?

R: The inherited diseases you get from your parents can be inherited by the parents too and it is possible if someone gives birth to 4 children maybe 2 out of the children.

I: Do you know anyone who cant see in your surrounding?

R: Yes, my wife and I are in the same position.

I: What led to your wife blindness?

R: My wife’s blindness; her parent had their sight intact………. Phone rang

I: You were talking about your wife’s blindness

R: Her parents had their sight intact and only God knows why because she was born blind but her blindness is partial and not total because total is someone who can’t see anything at all and partial is when you can still see some things but not completely.

I: So hers is partial

R: yes, then.

I: But now she can’t see at all?

R: Yes she can’t see again at all

I: And you said your wife is 47 years old

R: yes

I: aside from your wife, do you know anyone who can’t see with their eyes?

R: That cannot see? Yes because we are in the school of handicap so I have many of them around and many of them were born blind and some became blind at a point in their life maybe at the age of 30, 40 and some 50 years. May God have mercy on us anyone is not immuned from such events

I: Amen, So apart from the people born blind, what other thing do you think can cause or lead to blindness?

R: Measles, and in Yoruba land we have people that are always unnecessarily angry about people’s success academically, in business or career and this can lead to infecting people with blindness. I have a friend who works at the abattoir; he only became blind on one day when we was cutting meat, water splashed into his eyes and he became blind, his two eyes was gone and that was not an accident o, he just became blind like that.

I: What do you think people think about blindness?

R: Yoruba people says a prayer and its like this “may the lord not make us who is that”….. Like now if you come into the house and since I can’t see you, I will have to ask who to know who you are because I can’t see but if you have come and you decided to visit again tomorrow, next week or next month and I have mastered your voice to be able to recognize you

I: so that doesn’t mean if you are blind your life has ended?

R: If you get to my place you will see life in different dimension and remain hopeful again because I have children, wife and I have built a house. I have actually done and accomplish what people do as humans.

I: What do you think about blood donation or blood transfusion or donation for research? What do you think? Do you think you can participate in such?

R: you know you only give or transfuse blood to a sick fellow and I think they wont request blood for anyone that is whole or not sick

I: Yes

R: So if someone is sick and needs blood and test is carried out on my test and it is found out the blood matches then I can donate blood to the individual

I: Then along with collecting blood for the purpose of conducting research in the community to investigate for certain things in the blood. So what is your view on that?

R: I don’t have any bad thought about it

I: so if anyone approach you for you to donate blood for any form of research can you give your blood for such?

R: Yes I can give my blood

I: What do you think about like can you receive blood from people?

R: You mean like collect it from people?

I: Yes maybe you are in need of blood

R: Like?

I: God forbid bad thing o but maybe you need blood can you receive blood from someone?

R: You know when you are healthy you won’t need blood but if they recommend if I need blood but as old as I am I have never received blood of any kind before and I have actually prayed to God till I die not to have any reason to require blood transfusion

I: If a test was carried out on your blood can you accept the result that is obtained from the test?

R: Yes I will accept if it is my result

I: which one will you prefer to give out in these ones during research blood, saliva or stool?

R: I can give all but it depends on the required one by the researcher

I: Do you think there is any culture against this donation of these things in your community?

R: Maybe during our fasting period as Muslims because we don’t do such during our fasting period but aside that we can do that all season. But you know this world is tough and evil now because some people can collect it for some not nice purposes but asides that no one will resist giving blood.

I: What is your view about collecting people’s blood for research to test if the blood contains inherited genetic disease from the parents?

R: I don’t have an otherwise thought about it

I: So if you are approach to donate blood for research now, you will willingly oblige?

R: Yes I will give

I: What do you think about someone who took part in the research who won’t receive immediate benefit from the result of the research? Do you understand?

R: You for those things taken from my body?

I: Yes

R: I do not have any thing I think about it.

I: Like now I am conducting a research and you were a part of the research but I don’t have what I can give as a benefit immediately, will you still go ahead to participate in the exercise?

R: Yes I can do it now

I: You can do it

R: I will just think for example if I give people stprry about my life and if the person do and become grateful in the future I will just let them be.

I: So if you see the research as something that will benefit some other people

R: Yes I love to do and render help for other people in as much as it will help them. It’s just like sowing a seed of help because that individual can result to helping others too.

I: what do you think about these tests in Nigeria, do you think it is a nice thing to do in Nigeria?

R: Which tests are you talking about?

I: Like blood tests we are talking about

R: if it is not use for something evil then its it is a good thing and you know now in Nigeria many evil people takes a lot of thing from people for ritual purposes and as you are standing you alone know what you have in you and I only know what I have towards you in my mind. Only God can ascertain the thought of people’s heart.

I: Do you think it has any benefits for the community

R: Yes it has benefit because when we refuse to help people now what if later in the future we or our offspring are victims of these circumstances.

I: What can you highlight as challenges that can arise if someone wants to conduct this research?

R: People can be scared because their heart will be filled with questions like what does he want to use those things for?

I: How do we overcome this challenge you mention before carrying out these tests in your environment?

R: If you approach just one person it could be a little difficult.

I: So if I need people to trust me to allow me conduct this kind of test on them what way can we follow to ensure people trust us and eventually allow us to conduct the research?

R:The only way I think is to gather them and sensitize the people on the work and test. And also assure them of the safety and not that we want to take all their blood

I: You mean they should be well informed on all we want to do with the tests?

R: Yes, for example someone around 2013-2014 someone said he was asked to donate blood maybe at Adeoyo or UCH and people were what was given to you as compensation at least what you will take that will aid your quick recovery? And someone said there was a time when someone donated blood and after the whole exercise the fellow became unconscious.

I: So you think if we want to collect blood and we give them something for quick recovery you think that can encourage the turn out?

R: Maybe but I am not entirely sure.

I: Like now before carrying out this kind of research in the community who do you suggest I speak to before anything?

R: You will need to see the head of the community such as the chairman of all landlords or anyone who is there

I: But what about your own environment who do you think we can approach for such?

R: I am not sure about that or maybe on Fridays you can reach out to the Imams or the chairman landlord association.

I: Okay

R: you will inform them about the whole process of the work.

I: Do you think these genetically inherited diseases can be cured?

R: Hmmmmnnnnn, myself for example I am a Yoruba indigene, our parents are traditionalist and I must confess about inherited diseases, it is always difficult to cure such or else if God is interested but it takes time. But if it comes up naturally it can be cured easily than what was inherited.

I: Do you think the diseases inherited from parents can be prevented?

R: You mean to prevent inheriting these diseases? Well before we give birth if a man and a woman went for a test maybe that can prevent it but I think the chances are little.

I: what can we do about these inherited diseases from parent?

R: maybe when such individual visits a clinic and allows the health worker to flush out some of the blood and re-infuse some new blood into the fellow who is whole and healthy.

I: what is your view about data transfer, like what is your view about third party having access to your test results?

R: I am different because I don’t hide things

I: Because the researchers sometimes uses the results obtained from you for some other important works outside the original research. So what do you think?

R: I don’t have issues with whatever will help humanity because everyone needs help.

I:If a test is conducted on you now would you be interested in receiving results immediately?

R: Yes I will be interested o

I: Maybe if you were tested for a certain disease, will you be interested?

R: You know diseases are different and since the researcher didn’t put the disease there and if it was not wrongly diagnosed. For example someone who is not wayward or promiscuous, if diagnosed with HIV, you know such fellow could be curious and start thinking if there wasn’t a mistake somewhere. But I think the best is to listen to listen to the test results.

I: Maybe you were not battling with any disease but the result revealed you have the disease in your blood but will you be eager to know the result?

R: Yes me as a person will have interest in such results and if its something I can prevent then I will ensure I treat with the health care provider.

I: Before a test is carried out with you, what will you demand for from the person such as information?

R: I really wont demand anything because since you and I have been talking I can see you are here to ensure all is well with us.

I: So if you are told its for your benefit will you allow them?

R: Yes

I: Do you have any other information you can share with us on genetically inherited diseases?

R: No I have said all I know.

I: Thank you for your time, we appreciate it.
