# Supplementary material for: The social construction of genomics and genetic analysis in ocular diseases in Ibadan, South-western Nigeria
Source: PLoS One. 2022 Dec 1;17(12):e0278286. doi: 10.1371/journal.pone.0278286 (PMC9714877; doi:10.1371/journal.pone.0278286)
Supplement: S1 Appendix — (ZIP) [file pone.0278286.s001.zip › IDI 11 Male.docx]

**Keys**:

R- Respondent

I-Interviewer

I: I said at the beginning that I am doing a research on knowledge, attitude and perspective on ocular genetic research. Now, okay, are you listening?

R: Yeah, I am listening?

I: I will just like to know a couple of things, so feel free to answer as honestly as possible and I will be recording this session so I could transcribe and analyze later, I hope you do not mind?

R: not at all.

I: I will like to know if you can briefly explain to me how the visual impairment started or how it happened

R: Okay, actually I wasn’t born blind but lost my sight as a teenager.

I: Like how old were you?

R: Hmmmnnn, though I didn’t lose the both sight at the same time, I lost the right eye when I was 10 years old and then I didn’t know I was not longer seeing with that particular eyes so one day I came back from school and my mother just noticed I am not seeing with the particular eye again and for them to be sure of what they noticed or observed and this they did by closing the other side of the eyes and leaving the other one opened and that was when I discover that I could no longer see with that particular eye, so then I was taken to federal medical center in my state and after much delay I was taken referred to Kaduna after which I got to Kaduna national eye center and the doctor said there was nothing they can do and the best thing for me is to start using glasses to finish primary school and part of my junior secondary and I found out I was unable to see with the other eye too even with the lens and I reported to FMC again but taken to a clinic in Aba, and the Dr that examined my eye in Aba sent me back national eye center in Kaduna and they said the eye was a little bit and they don’t have the required equipment to carry out the surgery and they said they were on strike and there is nothing to do and based on ranking that was the highest eye care clinic in Nigeria and cannot refer me to another clinic or another hospital and they said my father should take me abroad and he asked where particularly but they said they don’t know but my uncle later discovered another Dr. who said he was a specialist in eye care that he studied in England but not a Nigerian, he was a Malian, the man said I had the hotness of the eye, that was how he described it. Actually, the first diagnosis they made at FMC was a retina detachment and cataracts and it was difficult for them to operate on it. The specialist was treating me and there was no improvement so I found out the man was just eating our money and I woke up one morning and I just told my father I am no longer going to that man again so they were making plans for me to go for surgery in Germany then and the hospital they contacted in Germany referred me to one of their branch in Lagos instead of coming to Germany and I was referred to Eye foundation in Lagos. Though they said the eye as already gone bad but they promised to try and see what they can achieve

I: Do you mean the other eye?

R: I mean the other eye was already bad even with the glasses, when we got to eye foundation and they discovered I have been going for treatment with someone and the man had given me medication which has further damaged the eyes because he didn’t understand the cause of the problem. My vision had already started going bad; this made them booked me for the first surgery at Eye foundation that lasted for about 7 hours which was followed by another surgery after 2 months and after that surgery they told me that the veins of my eyes are very weak and there is nothing they can do and I was advised since I was still young I can still go to school. And that was how the challenge began.

I: Okay, and all they said caused it was?

R: Retina detach and cataracts in both eyes but the cataract was successful thought the retina was bad and they did all they could to save the retina and another thing they did was the issue of the vein been weak.

I: did they mention anything about glaucoma?

R: I didn’t have glaucoma

I: So how did you feel afterwards?

R: Actually when I lost the first sight I didn’t feel much bad because I was still very young around 10-11 years and what can he think but all I noticed is I saw my father crying and I was like why was he crying? And when the second one started I was almost 16 and inside me I knew the eyes was already gone because while I was attending the FMC seeking treatment after the first one went bad and what I call carelessness now but after the second I told my younger sister my life has ended now and that I am completely dead o. when we were at national eye center Kaduna and they broke the news to us I was heartbroken and cried along with my father to extent that my father was so emotional that I had to console him afterwards, so that is just it.

I: Did you at any time attach any form of traditional belief to the cause of the blindness?

R: Come again

I: Like did you totally accept the diagnosis of the drs or maybe at some point you believed it could be as a result of some attack traditionally or witchcraft?

R: Okay, you mean Africa magic, right?

I: Yes

R: Actually I came from a well-educated home and the religion we practice we belief in what it says and though at some point we believed in it. My dad said that not everyone will see but its unfortunate his son is part of the ones who won’t see so I had to buckle up and go to school whether it is a spiritual problem or not and there is this popular saying in Nigeria, they say the village people and even till now I have not been bothering myself if it is village people or not, do you understand and I don’t believe about any spiritual anything because there was a practical detachment of retina.

I: what will you say about blindness being inherited?

R: It is possible because there are some families that blindness runs among them and they normally have issues with their sight but in a situation of someone who has glaucoma and retina detach could be due to nurturing the child which eventually leads to sight damage. But there are truly some family who has the disease of blindness running in their family and some it just happened naturally and there is this belief that when you have something to do with the blind such as marrying someone who is blind that the person could be blind or give birth to a blind child but that is not it.

I: Do you have a relative who is blind?

R: no, not at all, I am the only one with visual impairment but my father when he was alive he started using glasses but that is something sort of normal and common among people above 40 years of age in Nigeria. There is no person with blindness in my family.

I: Do you know of anyone who has an inherited eye disease?

R: You said?

I: Do you know of any friend or colleague who has an inherited eye disease?

R: When I was in special school where I met with some friends who are partially blind and I have not seen siblings who are all blind.

I: Thank you for the time so far we will be moving on to the genetic part of the study

R: The genetic what?

I: We will be moving to the genetic question of the interview

R: Okay, no problem

I: I will like to know what views are about blood donation for the purpose of research like if

someone come to you now and ask you to donate blood for a research on going on the eye and

they request you donate your blood to know the cause of blindness genetically. This could be beneficial to some people. So what is your view about such research?

R: I will definitely support it because of I have series of plans concerning issues like this to help do something to prevent blindness or treat the disease and I won’t just donate my blood if I am financially balanced I will also give money to support the research that is been carried out. I am saying I will donate my blood and other things that are required to conduct the research.

I: Okay, have you heard about genomic testing before?

R: I have not really heard about that and you are the 2^nd^ and 3^rd^ person I will hear this from.

I: that means you will be willing to partake?

R: Yes, I will be part of it and after this time we need to talk even and see how I can get involved. And if I may ask how can I get involved in this particular program. And what can I do to be part of this research?

I: I will tell you about that at the end of the interview and I will quickly wrap up the interview to get on that for you.

Then, something else happen in genomic research where we can take people’s blood sample to test for, for example in the case of a sickle cell if they it is found out that someone is AS they will advise the fellow not to marry a fellow AS because of their children and if we tell you the same thing with you and if we tell you the result from this research will be shared with a 3^rd^ party maybe funders and other research partners who may want to find out different thing using the gathered data will you agree to this? Will that affect your participation personally?

R: Yeah, it something that has to do with the sustainability of human development so for me I have no issue with that and as much as what they told me it will be used for is what they ended up using it for.

I: what do you think could be the possible challenges we can encounter doing this kind of research in a community with blind people? This is beyond you now as it is a broader context.

R: when you talk about the blind community I will like you to specify

I: for instance the special school for the blind now, we can call it a community of blind and also a general community too. What do you think about people’s willingness, do you think people will be willing or what do you think?

R: That could be a little bit challenging because in a situation where you are not educated or exposed you might see it as a big deal and they might not completely by into the research because so many people might think that you need their blood for ritual as Africans. For example where we have more of educated people, it could be achieved but you have to educate them to on why you need the sample because taking blood from someone without any explanation could raise a lot of questions.

I: In the school you went to do you thing many people will be willing to participate in this kind of research?

R: Yes, majority of them will be willing to take part.

I: Thank you very much for sharing your story and we assure you it is going to use for research purposes alone.

R: Thank you so much and I believe sharing my story and donating my blood will be for a good cause too. I wish you the best in your research

I: we will talk about the research some other time and I hope I get the text message you sent so I can call your friend if it’s not late. Thank you, bye

R: bye
