# Supplementary material for: The social construction of genomics and genetic analysis in ocular diseases in Ibadan, South-western Nigeria
Source: PLoS One. 2022 Dec 1;17(12):e0278286. doi: 10.1371/journal.pone.0278286 (PMC9714877; doi:10.1371/journal.pone.0278286)
Supplement: S1 Appendix — (ZIP) [file pone.0278286.s001.zip › IDI 12 Male.docx]

IDI with P-KAP

**I: Interviewer**

**R: Respondent**

I: Ok sir, please can you tell me your name, your age,

R: XXXXX

I: your age?

R: 63

I: where do you stay?

R: XXX, old Ife road

I: ok sir, I will like to know, what do you know about genetics and disease?

R: I don’t know very much about it

I: ok, do you think there are diseases that are inherited?

R: yes, I know

I: could you mention like a few of them

R: like blindness, mental problem

I: do you know of any blind person apart from yourself?

R: in my family?

I: not necessary from your family, may be your friends, family or those in the neigbourhood

R: I know many of them

I: about how old is the blind person that you know

R: like 50

I: so sir, for you what caused your blindness

R: the doctor said initially, it was cataract, then later to glaucoma,

I: what other cause of blindness do you know?

R: river blindness and the rest of it

I: so do you believe that blindness can be caused spiritually?

R: I don’t believe it

I: you don’t believe that? Ok and you don’t believe that blindness can be caused by something bad that somebody has done?

R: well, it depends, I mean

I: it depends on what sir?

R: it depends on how you look at it,

I: how do you look at it sir?

R: personally, I don’t believe it but people say so

I: so do you think blindness can be inherited?

R: quite alright

I: so what do you think about giving your blood for the purpose of research

R: hemm, in those days before I became blind, I use to donate blood at the blood bank, so

I: what about now, if we say, ok oo we want to conduct a research, and we will need you to donate a sample of your blood, what will you do,? Will you be willing to participate in such a research?

R: why not, if not?

I: so you don’t have any problem donating blood for research purpose?

R: I don’t have any problem

I: what about receiving the result of the blood test, what do you think about receiving the result of the blood test?

R: it is ok

I: now, if we say, between blood, saliva and stool, which will you be more willing to give for a research?

R: any one

I; why are you so open to giving anyone out?

R: it is not new now, is it new? I am use to giving it. You know I attend Jaja hospital in UI, and they often request for it,

I: that is for test

R; yes, for test,

I: but this one now we are doing a research we don’t even know whether, the research is not for you it is for the general public, but they are saying we need your stool, your saliva or your blood

R: there is no problem

I: please speak louder, I am recording it.

I: No problem sir, so sir in this community, are there cultural beliefs or religious beliefs about blood?

R: hemm, well, I should think so

I: can you share some with me?

R: heemmm, yes, some people say witches or wizards or so, I mean locally oo, they said they use to suck somebody’s blood, whether it is true or not, nobody can tell because it is not scientifically proved ooo but they say it

I: and this belief is it very strong in this community?

R; yes, it is very very strong

I: why did you say it is very strong?

R: because if someone die mysteriously, they will say he or she fights with someone and they will say that was the end or the witch confess that they have been chopping the body little by little and they’ve been sharing the meat part little by little

I: ok sir, I know I have already asked you and you said you don’t have a problem with it, so if I say we want to collect blood sample from you and may be everybody in this community, to know the genetic disease that they may have. What is your view about it?

R: yes, some people will respond and some people may not respond,

I: but you will respond?

R: haaaa, why not if not

I: and you will readily give us the blood or anything we ask for?

R: yes, anything

I: so what we tell you that we are going to do this research oo but it will not benefit you in anyway, it may benefit other people or generation

R: research is research, research is research

I: so you will still be willing to participate sir?

R: why not if not?

I: so in Nigeria, currently, do you think genetic testing is relevant? All this genetic test that we do to know if there are diseases in the blood. Do you think it is relevant in this our country Nigeria?

R: it is useful

I: why do you think so sir?

R: because it will assist the researcher to know what to do to know how to take care of the body

I: so do you think that genetic testing will be relevant to this community?

R: it will be relevant oo

I; so let’s assume we want to conduct a research like that in this community, what are the steps you think we need to do what are the things we need to do, if we say we want to conduct a research where people will have to give their blood or saliva among members of this community what should we do?

R:… hemmm well, except for if you go through hem any medical setting, like say clinic or any other places like that because people will not be willing to give all these things,

I: so we should go through medical settings, so what if we go through the community leaders?

R: I wouldn’t say because hemm.. maybe, if you go through the community leaders, it may be possible

I: but you don’t think it can be as effective as medical setting?

R: yes, that is it

I: so what are the challenges you think we may encounter from people?

R: they will not willing, that is the challenge

I; so how do we overcome that unwillingness?

R: the only way is that you go through medical setting, you know when they come for medical checkup or when they come to the hospital, if the doctors or nurses speak to them, come on so so date to meet our people then they will respond

I: what is your view about treating inherited diseases? Do you think they can be prevented, treated?

R: what is that?

I: inherited diseases?

R: hemm, they can be prevented

I: they can be prevented?

R: yes,

I: ok, so sir, now if we do that kind of research and we say we want to share the result with another set of people nit all, so we came to you we say we want tom do a research we took your blood, we say we want to check what is in your blood but now the result of that test we share it with some other group of researcher, would you still be willing

R: hahaa there is nothing bad in that ma

I: but before we share that your result with other researchers whom may use it for other purposes, would you like to be told, do you think we should tell you, or what if we don’t tell you is that ok by you or what do you think sir?

R: in as much as you’ve informed me now, I don’t feel there should be any problem. Not informing me can cause problem.

I: what kind of problem can it cause sir?

R: hem because in as much as I am not told, if I should know or hear about what has happened to anything that was taken from me. You know I won’t be very happy about it

I; so sir, would you like to know the result of your test? If we do any test for you would you like to know the result of it

R: it is better, I will be willing to know, and I will like to hear.

I: if for instance you find out that in your gene if there is a possibility of you developing a problem later in the future but you don’t have it now. Would you like us to tell you?

R: that is the only problem behind injecting, I mean taking blood and the rest of it. That is the kind of problem behind it.

I: what is the problem sir?

R: you know the needle or anything you want to use is not well sterilized, if it is not new or well sterilized it can cause can cause another problem in that person’s life

I; ohh no sir, apart from that needle now, the needle was well sterilized, and everything is fine but we found out that in their blood there is a disease inherit in your blood that you dint have now but there is a possibility of developing it in the future, would you like to know?

R: why not?

I: we should tell you?

R: yes

I: would you like people to identify you like when we want to give you your result and we say this particular result is for baba ooo, would you like it to be identifiable like that

R: no oo haaaa, ok

I: you will like it to be anonymous so that people will not be able to identify you

R:yes
I: so before we carry out a research like a genetic study when I come to tell you we are going to take your blood oo, we want to do a research for something something something, what kind of information would you like me to tell you that will enable you to participate in the study, what will you like me to tell you?

R: every possible information

I; like what sir

R: like what you want to do with it, like what is going to happen to the result

I: alright sir, thank you.
