# Supplementary material for: The social construction of genomics and genetic analysis in ocular diseases in Ibadan, South-western Nigeria
Source: PLoS One. 2022 Dec 1;17(12):e0278286. doi: 10.1371/journal.pone.0278286 (PMC9714877; doi:10.1371/journal.pone.0278286)
Supplement: S1 Appendix — (ZIP) [file pone.0278286.s001.zip › Male FGD Transcript.docx]

**FGD Recording**

(*I: Interviewer, P: Participants 1-6*)

**I**: I want to ask what you know about diseases in the blood, have you heard about it?

**P.1**: Diseases in the blood, we hear about it around; either on the radio or people talking about it, on the television news and even from the Hospitals.

**I**: What have you heard about it? What were you told about it?

**P.1**: They said there are some diseases found in the blood of people, it’s different from the diseases that befall a person by surprise or that is contacted from another person. But that these ones are found in the blood and live in the blood stream. So we are not new to this.

**I**: Okay, thank you Participant 1. Participant 2, have you heard about it?

**P.2**: I’ve heard about it.

**I**: Okay, can you give me examples of such diseases that are found in the blood and could be inherited?

**P.2**: People with diseases in the blood or such families, the disease in their blood are something that’s common. It’s almost impossible to not find one that has such disease but it varies. There are some diseases of the blood that when treated, either with pharmaceutical drugs or local herbs can be cured. But there are some other diseases of the blood which cannot be cured or if when cured will return to the affected person.

**I**: Thank you Participant 2. Do you think that asides from the diseases of the blood there are other diseases that people may inherit?

**P.2**: Ah, the diseases that people may inherit. There are some homes in which what is known as “*Warapa*” (epilepsy), you know it’s a disease?

**I**: Yes Sir.

**P.2**: Okay, so this disease is found in some families and there’s no child born to that family that won’t inherit the disease. This is because they inherited it from their forefathers.

**I**: Thank you Sir. Participant 3, asides from the diseases which Participant 2 has mentioned, do you think there are other diseases of the blood that one could inherit such that if the mother has it, the child could have it?

**P.3**: Emm…there are others.

**I**: I want you to mention some of them.

**P.3**: Among them are diseases like maybe diseases of the eye.

**I**: Diseases of the eye?

**P.3**: Yes. It could happen that the grandfather of someone or the grandmother of someone had a disease of the eye. Because there are some families where in their lineage it’s only the females that lose their sights and the males do not. So disease of the eyes is one of those inherited. Another one I know is one we could call mental illness. That’s all I can say for now.

**I**: Okay, thank you Participant 3. Participant 4, I want to ask you that asides the people you meet here, do you know others who have lost their eyes?

**P.4**: Quite well.

**I**: Quite well? These people, how old could they be?

**P.4**: From the ages of 5, 6, 7 different ages as to when they contracted the disease. And there are others who were born that way.

**I**: Such people of 5 years of age, what do you think caused it?

**P.4**: The reason is, when you investigate their families well you would find that there were others who had it or it could also be that the child is the first. Perhaps what the parents are used to eating or drinking and so the disease may have entered their blood.

**I**: So what you are saying now is that it is possible for blindness to be in the blood and be transmitted.

**P.4**: Yes

**I**: Thank you. Participant 5, I want to ask you sir, what are the other things that could cause blindness? Some of them have been mentioned, it has been said that it could be hereditary.

**P.5**: Among the causes of blindness is diabetes, which we know as the “sugary urine”. Also what we eat, such as, Starch, “*Lafun*” (Yam flour), “*Eba”* (Cassava flakes), Bread, these could cause blindness. And then what could cause blindness is when we stay in an area with lots of dusts or particles from where one may be working, such as, welding sparks or motorcyclists.

**I**: Thank you participant 5. Participant 6, I want to ask that asides from what Participant 5 has mentioned, do you believe that blindness could be caused by supernatural forces?

**P.6**: Some are the works of supernatural forces but other times it is not the work of supernatural forces, people may lie and deceive themselves. Then some are inherited and some are caused by high blood pressure. What we eat is also a cause of blindness.

**I**: I want to ask what your view about inherited blindness is.

**P.6**: My view about inherited blindness.

**I**: Yes, your view about disease causing blindness in the blood that is heritable.

**P.6**: I think it happens that way. Because if we look at it, we may meet someone with a disease of the eye, if the family is studied properly, we would find that it could have been inherited.

**I**: Thank you. Participant 1, I want to come back to you. What is your view on inherited blindness?

**P.1**: Inherited blindness isn’t an uncommon thing. It can be commonly inherited, and many people can call it a hereditary problem, one that is common in a lineage. Just like Participant 2 or 3 had said that it could be the grandfather or grandmother of a person that this disease had come. So it’s quite prevalent.

**I**: Thank you Participant 1. Participant 2, what is your understanding on blindness been hereditary?

**P.2**: It’s not a new thing and when it happens it happens. And when it happens, we should find a way in which the affected can be a significant member of society. Such as, taking him to a school for the blind because in some cases a blind person might be more useful than someone with both eyes. So, it’s not a new thing and when it happens it happens, and we should accept that it is Gods will, that’s how God wanted it to be and it is. So, it is not that we should be depressed or that that’s the end for us. So that’s how it is.

**I**: Thank you, Participant 2. Participant 3, I want to ask you, do you agree that blindness could be caused by supernatural forces?

**P.3**: I agree to it and I also disagree to it.

**I**: Okay, explain.

**P.3**: Firstly, why I agree to it is that it could be spiritual forces because anything can happen. Secondly why I disagree to it is that it is in the Book of God, and then it could also be self-inflicted. Because anything can happen, anyone could make a mistake and cause what he isn’t supposed to and affect his wellbeing. So, these are the reasons why I agree and I disagree that it could be caused by supernatural forces.

**I**: Thank you Participant 3. Participant 4, do you agree to such beliefs that blindness could be caused by supernatural forces?

**P.4**: Ehn… I believe it and I also do not believe it because if we look at those of us who are blind we can say whether it is caused by supernatural forces or not because each person knows how the affliction came about so whether it was caused to him by supernatural forces or not. Because if we look at it, if someone is suffering from measles and it’s not properly treated it tends to take something from the person. Also, when we look at it, it could be someone who is troublesome and got in a fight or something and during the fight it’s possible that someone hit him with something. Each person knows what process may have occurred for the affliction to happen to him. But I believe that even the supernatural forces, a leaf does not fall without God knowing when it did, so God allowed it if it were the works of supernatural forces.

**I**: Thank you Participants 4. I want to ask you, Participant 5, what is your take on giving blood samples for a research? We want to make a research and request for your blood sample, what’s your take on it?

**P.5**: My take on leaving a blood sample. If one wants to give a blood sample. Does it depend on the amount of blood in the person’s body that will make him know how much blood he’s leaving or …I don’t understand.

**I**: Okay. What if it’s a tablespoon of blood? If it’s a tablespoon of blood that you’re asked to give and not a whole lot of blood that we want to investigate, we want to know – you know these heritable diseases are in the blood so we want to know what causes it in the blood and so we are requesting that you leave a blood sample for research. What is your opinion on something like that?

**P.5**: I think it is okay. Because if they don’t use – if they don’t take such steps it is possible that they don’t know the type of thing that is present in the blood. But for them to know the kind of things in the blood is why they request a blood sample, so they may know what is in it.

**I**: Thank you Participant 5. Participant 6 I want to ask what is your take on giving blood sample for research.

**P.6**: Giving blood samples for research is good. If blood samples aren’t provided, how will one know what is in his blood? There is nothing bad in one giving blood sample for research.

**I**: What is your opinion on using such findings – for instance the research is completed on the sample of blood, the result found is shown to you. What is your opinion? Will you be happy to know the results or would you be less concerned?

**P.6**: It should be of concern to the person. For example, if it were me who left a sample of my blood, I should know the kind of disease that’s in my body which the result will reveal.

**I**: Thank you Participant 6. Participant 1, I’m back to you again. You as a person, if we were to conduct a research, which would be more convenient for you or which would you prefer to leave behind; blood, saliva or faeces?

**P.1**: Em, you know the doctor who is to conduct it would know which sample would be best to investigate.

**I**: Let us assume they could find the results using either of the three, you as a person which is more convenient for you to leave behind?

**P.1**: There is no sample I can’t leave behind.

**I**: So there isn’t any that could be inconvenient for you to provide

**P.1**: Yes

**I**: Thank you Participant 1. Participant 2, which would be more convenient for you? Or which would you prefer to leave behind among these three?

**P.2**: Ah, is it to spit that’s a problem? Or **laughs out loud** It won’t even cost me anything **background laughter** How many seconds will it take **laughs out loud**.

**I**: Thank you Participant 2. Participant 3, how about you? Is it blood or saliva or faeces?

**P.3**: There isn’t any that’s inconvenient.

**I**: There isn’t any that’s inconvenient?

**P.3**: Yes

**I**: Thank you. Participant 4, which is most convenient for you?

**P.4**: Ah, it’s saliva oh.

**I**: It’s Saliva, thank you. Participant 5, which is most convenient for you?

**P.5**: It’s saliva; quickly it’ll be replaced in my mouth. **Background laughter**

**I**: Participant 6, which is most convenient for you?

**P.6**: The saliva is the easiest; it will quickly be spat out.

**I**: Thank you. So what you are saying now is that saliva is the easiest to come by that’s why it’s convenient for you. Okay, I understand. Thank you very much.

Participant 1, I want to ask is there a culture or belief that inhibits people from leaving blood samples in the society you live in?

**P.1**: Ehn, well. Belief, there are some people, some religion such as Christianity where they do not believe in leaving blood samples. So much that they would prefer to die than leave their blood sample. So, the issue of blood sample, some people – **Interviewer interrupts**

**I**: I’m asking in your society where you reside. Could it be that their culture is against such?

**P.1**: No oh, which culture would be against such? No

**I**: Thank you. Participant 2, is there a culture or belief in your society of residence that has a negative view on leaving blood samples?

**P.2**: Umm… we can’t say it has a negative view on it and we also can’t say it doesn’t have because there are a multitude of things that has come to be in the world of today. One could leave a blood sample and it turns out that there might be another thing to leaving the blood sample that may be catastrophic. But if we do as people, the society we live in has no negative view on leaving blood samples. So I haven’t seen anyone who left a blood sample. That’s how it is.

**I**: Thank you. Participant 3, I want to ask you what your view is on leaving your blood sample for research and you as a person, would you be willing to leave your blood sample for research.

**P.3**: Em, I would be willing and I also won’t be willing on the other hand.

**I**: What’s the reason?

**P.3**: The reason is that you know that where Nigeria is today, the person that is well fed is the one who could leave a blood sample. So a person who is lean or doesn’t have weight, one like me – **Interviewer interrupts**

**I**: What I’m trying to explain to you is; this blood sample we are talking about is not the quantity of a keg, it’s merely a tablespoon.

**P.3**: I understand.

**I**: That’s the quantity you are leaving behind.

**P.3**: I understand but I may interest to leave a blood sample if God makes it convenient for me.

**I**: Thank you. For this research we are conducting, if the result of the research is not useful for you at this time but for others, would you still be willing to leave a blood sample?

**P.3**: Em… I will do it.

**I**: You will leave a sample. Thank you Participant 3. Participant 4, if you are not going to benefit from the research but others will, would you still be willing to leave a sample of your blood for such research?

**P.4**: Ah, before my blood is taken I’d firstly like to know what’s in my blood. Maybe there is something there or not. You know to leave a blood sample depends on ones feeding. Because when you mentioned a tablespoon, before the tablespoon of blood taken is formed again in the body **scoffs** it’s not within a week oh!

**I**: But you know there is 5 litres of blood in our bodies.

**P.4**: 5 later of blood?

**I**: Litre –

**P.4**: Ehn, 5 litres of blood–

**I**: –that is in our bodies. And if we take a tablespoon, you know there’s still a lot left.

**P.4**: Ehn, what each person eats is what gives them blood. You can’t say whether – **brief background contribution** – Ehen! What if someone hasn’t eaten since morning and doesn’t know what he would eat at night, maybe “*Garri*” (Cassava flakes) **laughs** *Garri* that depletes blood.

**I**: Thank you. Participant 4, before we leave I want to ask if we do a research on your blood sample would you like to receive the result?

**P.4**: I am interested. I want to know as well what the blood looks like.

**I**: Thank you. Participant 5, if we take your blood sample and investigate would you like to know about the results?

**P.5**: Ah, so why did I leave the blood sample? I want to know that’s why I left a sample.

**I**: Okay. What if we use that blood sample to run a research in which you would immediately benefit from or may not benefit from at all would you still be interested in leaving a sample of your blood?

**P.5**: As long as the research is being carried out and those taking the blood are not using it for something else. I will leave a blood sample.

**I**: Thank you. Participant 6, I want to ask you what your opinion about treatments of inherited diseases is.

**P.6**: I don’t understand the question.

**I**: What is your opinion on treatments of inherited diseases? Do you think it is curable?

**P.6**: Ah, I think it’s curable, it’s curable na if it is monitored from the start. You know in the old days, for example, there were no immunisations. In some homes they give birth to “Abiku” (stillbirth) children but now that there are various immunisations, in such homes they no longer give birth to such. You know that’s monitoring it.

**I**: Yes, thank you. Participant 1, I want to ask you what your opinion on treatments of inherited diseases is.

**P.1**: My opinion is that it is good because if the inherited diseases are monitored they would reduce in our society. So it’s good. My opinion is that when these diseases are treated they will reduce in our society.

**I**: Thank you. Participant 2, I want to ask that do you think all inherited diseases can be treated.

**P.2**: Ehn… You know that where science has reached as of today. There is almost no disease that can’t be treated now. So there isn’t anything that can’t be treated if you want to treat it and treat it well, there isn’t anything that can’t be treated in my point of view, that’s my perspective.

**I**: Thank you. Participant 3, I want to ask you do you think we can prevent such diseases.

**P.3:** Do you think…?

**I**: These diseases that are inherited even before they occur, do you think they can be prevented?

**P.3**: Yes.

**I**: What ways can we employ to prevent it?

**P.3**: Em… firstly, if God says it can be prevented it would be prevented. Also, in the case proper self-care, firstly, individual treatment that’s the first. Secondly, about science according to what Participant 2 has said, how far civilization has come. There is science and there are various scientific ways in which doctors practice and it’s possible to gain more knowledge to treat it.

**I**: Thank you, Participant 3. Participant 4, what are the ways in which we could prevent hereditary diseases.

**P.4**: Ways in which a person can prevent hereditary disease is what the other participants have mentioned. Maybe if someone is afflicted before it gets severe, if it is quickly diagnosed and the person taken to a hospital, there is a probability that it quickly get healed. Compared to when the disease becomes severe and the person is kept at home, it may take time before it gets healed but when it’s discovered early and the person is taken out its possible it reduces.

**I**: Thank you Participant 4. Participant 5, I want to ask you what is your opinion or what do you think about showing the results of your blood sample to a third party? Maybe it was me who took your blood sample and I’ve shown you the result and I went ahead to show a doctor, third party or someone else carrying out a research relating to what we are doing. Are you satisfied with it or you don’t care.

**P.5**: Err… I’m satisfied because thorough research is on-going in order to know what it is about so as to get a valid result. I’m satisfied.

**I**: Thank you Participant 5. Participant 6, what is your opinion about such? That the result of your blood sample is shown to a third party. Asides I that took the blood sample and have told you the result, if I show a third party maybe they are also carrying out a research. Are you okay with such?

**P.6**: I don’t think there’s anything wrong with that. I’m okay with it.

**I**: Thank you. Participant 1, are you okay with such? That the result of your blood sample is shown to a third party who is also carrying out a research.

**P.1**: Ehn, we are doing so to help each other. There is nothing wrong with it.

**I**: Thank You. Participant 2, what is your opinion?

**P.2**: When I don’t have AIDS **chorus background laughter** or why should I get scared that my blood sample is being shown to a third party. It won’t scare me, infact I would stand gallantly.

**I**: Thank you participant 2. Participant 3, is your opinion different from what the others have been saying?

**P.3**: There’s no difference.

**I**: No difference?

**P.3**: Yes

**I**: Which means you are also in support?

**P.3**: Yes

**I**: Thank you. I want to ask Participant 4, if we are to carry out a research relating to taking blood samples, what are the explanations you would like to hear in details? What would you like to know about the research before we commence?

**P.4**: The explanation I would have to know is that I who would be leaving a blood sample, hope nothing would happen to me. After I have left a blood sample, before I would have to leave my blood sample. Either they will want to use it for someone else or it would be used to carry out a research for me myself. Hope nothing will happen to me. And how long will it be after leaving my blood sample for someone before I recover.

**I**: Thank you. I want to ask Participant 5. What are the explanations you need to know before you agree to participate in the research? What would you like to be explained to you in details?

**P.5**: What I would like to know is that the blood taken for a test. I’d like to know the results, what are the things present and what is missing? What are the things that ought to be there and what ought not to be there? And then what could possibly show in the future so I could know on time what decisions to be made about it.

**I**: Thank you Participant 5. Participant 6, what explanations would you like to know in order to be like “When these things are explained to me, I would want to participate in the research”?

**P.6**: Like for me oh, what I would first ask is if I leave this blood sample for the research, if they find a disease in my blood is there any assistance to treat me for the disease there to be cured? Are you clear with my explanation? So if they say there would be such, I’ll agree to carry on the research. And if they say there would not be such, there’s nothing I can do about it.

**I**: Would you still agree?

**P.6**: I would agree but I would be happy if they tell me what they found in the test. At least if they cannot take care of me then let me be aware of the steps I’d take on time in order not to ruin my future of that of my children.

**I**: Thank you Participant 6. Participant 1, what kind of explanations would you like to hear before you participate in this kind of research?

**P.1**: Ah. Ehn, the explanations I would like is what others have been saying that this blood sample you are about to take hope it won’t have a negative effect on me. Then after it has been collected will I be told if I have a particular ailment or something in my body. And if there is, will I be helped in curing it? That’s what I’d like to know. And my blood sample they’ll be taking, hope it’s not that they want to use it for something that is not good.

**I**: Thank you. Participant 2, let me ask you if there is anything you would like to say that’s different from what others have been saying.

**P.2**: Em… if you want to draw blood from my body, surely I would ask for your doctor’s report, that is it true you are a doctor? And did you truly come – like now that you claimed to come from UCH. I would like to know that truly you came from UCH and that you are not fake or you are original. That’s the first one. Number 2, I’ll also ask that you provide a police report and the authority you received from the Chief of police to undertake this work because blood is life. So I would ask for a police report. Then who gave you the authority from the community to undertake such work, the person should give a note from his side, which I will request from you. Then I’ll also ask you that after you’ve come and drawn my blood, what you will give me to use or to eat that will recover my blood to my body without delay. For example, milk, Maltina, because that can restore the lost blood quickly. So I would first ask you, and if it happens that among the things I’ve requested, milk or Maltina, if you don’t have any, I cannot allow you take my blood. That’s how it is.

**I**: Thank you, Participant 2 for the explanations. Participant 3, What explanations would you like for you to participate in this research?

**P.3**: What I would like to what….?

**I**: What sort of explanations would you like to hear before you participate in such research and leave a blood sample?

**P.3**: Okay, hmm… just as Participant 2 has explained, so that’s what would be on my mind to ask.

**I**: Thank you very much Participant 3. I now want to ask all of you, is there any other explanations or things you want to say to buttress on the issue of the research on inherited diseases. Maybe there are some things you will like to add. Asides from all you have said.

**P.5**: What I have to say is that..

**I**: Thank you, Participant 5.

**P.5**: These types of inherited disease, for example, I that lost my sight and know that it is Glaucoma. This Glaucoma it seems it is hereditary but it will be good for one to monitor his child and conduct various tests. So if various tests are done they won’t be able to inherit the disease. And if this is done from the start, it will be avoided. Such things can make for the eradication of inherited diseases in the society.

**I**: Thank you Participant 5. Does anyone have any other contribution? In the absence of any other contribution, I am grateful to all of you for the time you have taken out for this interview; Participant 1 I thank you, Participant 2 thank you very much. Participant 3 thank you very much, Participant 4 thank you very much, Participant 5 thank you very much, Participant 6 thank you very much. May God grant you wellbeing.
